# Implementation and evaluation of personal genetic testing as part of genomics analysis courses in German universities

**DOI:** 10.1186/s12920-023-01503-0

**Published:** 2023-04-05

**Authors:** Tamara Slosarek, Susanne Ibing, Barbara Schormair, Henrike O. Heyne, Erwin P. Böttinger, Till F. M. Andlauer, Claudia Schurmann

**Affiliations:** 1 Digital Health Center, Hasso Plattner Institute, University of Potsdam, Prof.-Dr.-Helmert-Str. 2-3, 14482 Potsdam, Germany; 2 Hasso Plattner Institute for Digital Health at Mount Sinai, Icahn School of Medicine at Mount Sinai, One Gustave L. Levy Place, New York, NY 10029 USA; 3 Institute of Neurogenomics, Helmholtz Zentrum München – German Research Center for Environmental Health (GmbH), Neuherberg, Germany; 4 Institute of Human Genetics, Klinikum Rechts der isar, School of Medicine, Technical University of Munich, Munich, Germany; 5 Department of Neurology, Klinikum rechts der Isar, School of Medicine, Technical University of Munich, Munich, Germany

**Keywords:** Genomics education, Personal genotyping, Personalized medicine

## Abstract

**Purpose:**

Due to the increasing application of genome analysis and interpretation in medical disciplines, professionals require adequate education. Here, we present the implementation of personal genotyping as an educational tool in two genomics courses targeting Digital Health students at the Hasso Plattner Institute (HPI) and medical students at the Technical University of Munich (TUM).

**Methods:**

We compared and evaluated the courses and the students’ perceptions on the course setup using questionnaires.

**Results:**

During the course, students changed their attitudes towards genotyping (HPI: 79% [15 of 19], TUM: 47% [25 of 53]). Predominantly, students became more critical of personal genotyping (HPI: 73% [11 of 15], TUM: 72% [18 of 25]) and most students stated that genetic analyses should not be allowed without genetic counseling (HPI: 79% [15 of 19], TUM: 70% [37 of 53]). Students found the personal genotyping component useful (HPI: 89% [17 of 19], TUM: 92% [49 of 53]) and recommended its inclusion in future courses (HPI: 95% [18 of 19], TUM: 98% [52 of 53]).

**Conclusion:**

Students perceived the personal genotyping component as valuable in the described genomics courses. The implementation described here can serve as an example for future courses in Europe.

**Supplementary Information:**

The online version contains supplementary material available at 10.1186/s12920-023-01503-0.

## Introduction

Decreasing genome sequencing costs, new technologies, and an improved knowledge of the human genome brought genomic research to a level of application in life sciences and medicine that exceeds expectations from three decades ago when the Human Genome Project was launched [1]. Translation of genomic research towards an application in clinical care is continuously progressing, with the aim of improving diagnosis, disease prevention, risk prediction, as well as drug efficacy and safety assessments [2–6]. An understanding of data science has become a requirement for genomic researchers [1]. In addition, the interest of European citizens in direct-to-consumer (DTC) genetic testing in a non-clinical setting is high, even though genetic education and understanding is low in the general population [7].

To train a new generation of experts at the interface between computer science and medicine, the Digital Health (DH) Master’s program was initiated in 2018 at the Digital Engineering Faculty of the University of Potsdam, the Hasso Plattner Institute (HPI) [8]. In 2020, we introduced the Analyze Your Personal Genome (AYPG) course that incorporates voluntary personal genetic testing (PGT). Its aim is to enhance the understanding and to develop a sensitivity towards genetic testing by affecting the students on a personal level. The underlying rationale is based on self-determination theory: the students’ interest in the course content and their motivation for engaging in the course shifts from extrinsic motivation driven by grading to the intrinsic motivation of wanting to learn about their personal genomic background [9]. In addition to increased motivation, we aimed for enhanced compassion with patients and individuals undergoing PGT. Because DH students have heterogeneous backgrounds, a particular challenge was to design a course that provides learning opportunities to everyone, while ensuring that students take informed decisions whether to undergo PGT, regardless of their previous knowledge.

In response to the rising application of clinical and DTC genetic testing, the necessity of a profound genetic education in medical training was already recognized more than a decade ago [10]. At Tufts University and Stanford School of Medicine, discussions about introducing PGT into genomics courses to improve learning outcomes and motivation began in 2008 and 2009, respectively, and, after careful consideration, resulted in similar course concepts including voluntary genetic testing [11, 12]. At the Icahn School of Medicine at Mount Sinai, New York, personal genome sequencing was first applied in medical training in 2012 [13]. Strategies and results of these three schools were summarized and discussed by Garber et al. [14]. Briefly, several schools aimed to improve the genomics education for healthcare providers by adding an innovative and engaging component to their curricula, such as personal genotyping or working with anonymized or donated genomic data. Enhanced educational outcomes of students participating in PGT were indicated in studies accompanying the courses at Stanford School of Medicine and Icahn School of Medicine at Mount Sinai [15, 16].

Next to medical schools, PGT is increasingly applied in the genomics training of prospective pharmacists, with a special focus on pharmacogenomics. At the Eshelman School of Pharmacy at the University of North Carolina and at the University of Pittsburgh, voluntary genotyping is offered to advanced pharmacy and students in the Doctor of Pharmacy (PharmD) program to increase the understanding and acceptance of pharmacogenomics in the clinical context [17–19]. The classes included up to 145 students and genotyping was conducted by a DTC company. At the University of Florida, a smaller number of third-year PharmD students could undergo PGT of selected pharmacogenomic markers as part of the elective pharmacogenomics class [20]. The only published genomics course including PGT for undergraduate science students rather than medical or pharmaceutical students took place at the Brigham Young University [21]. Here, PGT was offered as part of the Advanced Molecular Biology and Genomics course. The students received their personal genotyping data not during, but after completion of the course, assuming that the students’ motivation would be increased by undergoing genetic testing. At the "Bring Your Genes to Cal” course at UC Berkeley, undergraduate students were offered to receive genetic information regarding three non-disease-causing variants. In 2010, upon discussions with the California Department of Public Health, the university decided not to return individualized results anymore [22].

Many of the offered courses in the US rely on genotyping performed by private DTC companies [12, 15, 17–19, 21]. In Europe, there is no uniform legislative framework covering DTC genetic testing across the 27 individual member states [23]. In Germany, genetic testing is regulated by the Genetic Diagnostics Act (Gendiagnostikgesetz, GenDG) [24], which requires testing to be carried out by medical doctors, following written informed consent; in case of preemptive testing, genetic counseling by certified medical doctors is required. Therefore, the GenDG does not allow for delivery of clinically relevant results within a DTC genetic testing framework [25]. However, it is allowed to obtain raw genetic data. Additionally, genetic testing for research purposes is exempt.

The present paper describes the implementation and evaluation of the AYPG course at HPI in Potsdam, Germany. Moreover, the AYPG course is compared to the Genomic Medicine (GM) elective course at the School of Medicine of the Technical University of Munich (TUM), which, to our knowledge, was the first course at a German university to include a PGT component in 2017. The AYPG and GM courses were evaluated using questionnaires to assess the students’ attitudes concerning PGT and its usefulness as part of an educational program.

## Materials and methods

This section describes the implementation of the AYPG course and the course questionnaires.

### Course implementation

**Fig. 1:**
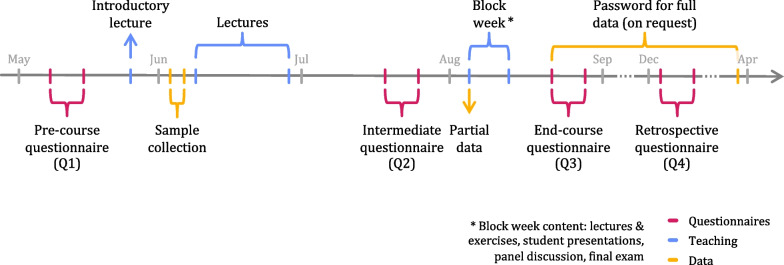
Timeline of the analyze your personal genome course. Magenta items indicate questionnaire periods, blue items teaching activities, and yellow items events related to data

The elective AYPG course was offered to DH Master’s students at HPI. After a course briefing at the beginning of the summer term 2020, covering relevant information about the course and establishing the voluntary nature of the PGT, 16 students in their second year of Master’s studies participated in the course. The course consisted of four three-hour remote lectures throughout the semester and a five-day in-person block course in August 2020 (Fig. 1). A second iteration of the course was conducted in summer term 2021.

We structured the learning outcomes for the AYPG course according to Kraiger et al. [30] into cognitive, skill-based, and affective learning (Table 1). While cognitive learning outcomes mainly refer to the accumulation and organization of knowledge, skill-based learning outcomes are related to the acquisition of skills and automaticity, and affective learning outcomes are related to attitude and motivation. The course started with an emphasis on cognitive learning outcomes, including the basics of human genetics, pharmacogenomics, and clinical genomics, preceding the practical modules. In the second part, the focus was set on skill-based learning outcomes in combination with knowledge transfer.

**Table 1:** AYPG course schedule containing learning objectives: the course was structured into 12 sessions of three hours

Session number	CW	Session topic	Learning outcomes		
			Cognitive	Skill-based	Affective
1	22	Introduction	General overview German Genetic Diagnostics Act (Gendiagnostikgesetz)		Understand context and decisions facing patients, researchers, and clinicians
2	25	The Human Genome	Basics concepts of molecular genetics/the human genome Genetic diversity and genetic variation Population structure		
3	26	Pharmacogenomics	Basic concepts and examples of pharmacogenomics	CPIC guideline interpretation	
4	27	Genomic medicine	Effects of genetic variations, clinical relevance		
5–6	32	Genotype–phenotype relationship	GWAS and GWAS meta-analyses: Basic concepts, examples	Hands on exercise using PLINK Explore GWAS summary results using FUMA and MAGMA	
7	32	Technical Aspects	Genotyping & genome sequencing techniques		
8	32	Genomic Research, Annotation	Overview genomic databases/tools and data resources used in annotating and interpreting a personal genome	Hands on exercise: Exploration and interpretation of selected association results	
9	32	Direct to consumer testing	Basic concepts, Polygenic risk scores		Understand ethical, legal and social implications of genetic testing
9	32	Disease Risk	Interpretation of SNV findings (effect size, causal vs. not, multi-allelic models) and the limitations thereof		
9–10	32	Polygenic traits and diseases	Basic concepts, Polygenic risk scores	Calculate polygenic risk score using the openSNP dataset	
10	32	Ancestry	Population structure in genomes and basic concepts (MDS)	Infer genetic ancestry of a genome from the openSNP dataset	
11	32	Genomic Research, Annotation		Exploration of selected personal genomic data Analyze found SNVs associated with disease risk in the context of public databases, literature, and other resources	Discussion and Presentation
12	32	Q&A with Human Geneticists			Discussion Review current understanding of how patients respond to genetic testing results emotionally and behaviorally

The first four sessions were held as separate lectures during the semester, the last 8 sessions were part of a block course at the end of the semester

After the introductory lecture, which included information about the risks and challenges of genetic testing as well as ethical aspects, students could choose to participate in the free-of-charge PGT. Additionally, a course information sheet and consent form were provided to ensure informed consent (Additional file 1). Students had to pass a quiz to make sure they had read and understood the course information sheet. In case HPI students had questions regarding their genetic test results, they were pointed towards existing external genetic counselling offers accessible to them. A study coordinator and honest broker who was not associated with the course coordinated the genotyping process, including communication with students, obtaining consent, collection of samples for PGT, and handing out the pseudonymized data to the students. The honest broker had no access to PGT information and researchers conducting quality control of the genetic data worked with pseudonymized data. The course instructors did not know which students participated in the PGT and which did not. Personal data was processed in compliance with the EU General Data Protection Regulation and the Brandenburg Data Protection Act. The described genomics course with PGT opportunity was approved by the ethics board of the University of Potsdam with application number 17/2021.

Genotyping was conducted using the Illumina Infinium Global Screening Array (GSA v3-MD). Throughout the course, only selected single-nucleotide variants (SNVs) were accessible to the students: specific pharmacogenomic markers, SNVs determining the carrier status of rare diseases with recessive inheritance patterns, and backbone SNVs not suitable for determining the individual risk of rare monogenic diseases and limited to assessing risk of common multifactorial traits or diseases (Additional file 2). The full PGT information was encrypted and password-protected. Only after successful course completion, students could request the password to their own complete encrypted data from the honest broker. To obtain additional large genotyping data sets, open-access data from the openSNP database was downloaded using the opensnp-cohort-maker pipeline [26, 27]. The final openSNP data set included 509,712 variants in 3,581 individuals. Furthermore, students who chose not to participate in the PGT component of the course or who chose not to analyze their personal genetic data during the course were assigned anonymous openSNP data for analysis.

### Course questionnaire

To evaluate the course and to assess the impact of the course on students’ attitudes towards genetic testing and analyses, we established questionnaires that were completed at four different time points: a pre-course questionnaire (Q1) after the introductory course, an intermediate questionnaire (Q2) before working with one’s own data, an end-of-course questionnaire (Q3) after working with one’s own data, and a retrospective questionnaire (Q4) about three months after completion of the course (Fig. 1, Additional file 3).

The overall questionnaire design was inspired by the questionnaires used to evaluate a course including genome sequencing at the Icahn School of Medicine at Mount Sinai [16, 28]. The first iteration of the Q1, Q2, and Q3 questionnaires contained questions about the motivation to participate in the course, the usage of personal genome data, usefulness of array-based genotyping data in clinical practice, usefulness of genome sequencing data in clinical practice compared to array-based genotyping data, and opinions on genetic analysis legislation, specifically the German GenDG. A Human-centered design thinking expert from the HPI was consulted to help with the design and implementation of the questionnaires as well as usability testing.

After the first three questionnaires were conducted at HPI, the survey was re-designed and shortened considerably, in coordination with co-authors from TUM. This re-design was conducted to make the questionnaire more accessible and increase participation rates at HPI and TUM, especially when the survey was only administered remotely. Instead of Likert scale questions with several sub-items and open-ended questions, much simpler questions with fewer answer options were introduced, focusing on the motivation to participate in the course and participation and attitudes regarding genotyping and data analysis in the course context. All questionnaires following the first three HPI questionnaires were adapted to this revised version.

To foster truthful answers, the questionnaire invitations and introductions clearly stated that participation is completely voluntary and anonymous. At HPI, pseudonymization was implemented with the help of the honest broker, enabling the anonymous connection of single students’ responses across questionnaires. Students agreed to the complete anonymization of their data after the last questionnaire by deleting the mapping of names to pseudonyms.

The questionnaires were created with SoSci Survey [29] and were conducted with students of the AYPG course in the summer term 2020, the TUM GM course in the winter term 2020/21, and the AYPG course in the summer term 2021. Additionally, a slightly adapted version of Q4 was conducted at TUM for all past GM classes (Q4’). HPI questionnaires were conducted in English, which is the teaching language of the DH curriculum, TUM questionnaires in German.

### Statistical analyses

Questionnaire answers were exported from SoSci Survey in CSV format and analyzed using R 4.2.1. Responses were compared in two dimensions:

First, HPI and TUM responses were contrasted using Fisher’s exact test (stats::fisher.test; stats v4.2.1). For effect sizes, Cramer’s V statistic was computed (lsr::cramersV; lsr v0.5). Effect sizes were interpreted according to the standard interpretation [29].

Second, longitudinal differences in questionnaire responses were assessed over the course (each iteration of the course was analyzed separately). For pseudonymized questionnaires, as conducted at HPI, paired categorical Likert-type responses over the course were compared with Wilcoxon’s Signed Rank test (stats::wilcox.test with option paired = TRUE). The *r* value was used as effect size (rstatix::wilcox_effsize; rstatix v0.7.2). 5-point Likert scale responses from long-version questionnaires were simplified by grouping them together regarding whether they indicated agreement (value of 1) or disagreement (value of − 1) while keeping neutral answers as a third category (value of 0). Paired binary responses were assessed using McNemar's test (stats::mcnemar.test), using odds ratio as effect size (DescTools:: OddsRatio; DescTools v0.99.47). Completely anonymous questionnaires, as conducted at TUM, could not be matched longitudinally to individuals; therefore, responses over the course were compared with Fisher’s exact test.

The code used for statistical analyses is available on GitHub (https://github.com/tamslo/sosci-questionnaire-evaluation/blob/main/genomics-lecture-specific/analyze-data/complete-analysis/CompleteAnalysis.Rmd).

## Results

This section compares the AYPG and GM courses and presents the main questionnaire results. In total, 13 questionnaires were conducted (Additional file 4): Q1 to Q4 for HPI 2020 (16 course participants), HPI 2021 (16 course participants [15 during the block week]), and TUM 2020/21 (10 course participants [8 in the block week]); as well as TUM Q4’ (102 course participants before 2020/214). Most questionnaire results in the following sections focus on Q4 and Q4’ questionnaires (HPI 2020: N = 10; TUM 2020/21: N = 6; HPI 2021: N = 9; TUM before 2020/21: N = 47).

### Course content and interests differed between student populations

The AYPG course at HPI, in particular the implementation of the PGT component, but also parts of its content and structure, was inspired by the GM course, which was first implemented at TUM in 2017. Nevertheless, the curriculum of each course was optimized for the different student populations and learning objectives (Table 2).

**Table 2:** Comparison of existing genomics courses in Germany

Course	Analyze your personal genome (Hasso Plattner Institute, HPI)	Genomic medicine (Technical University of Munich, TUM)
Student population	Second year Digital Health Master’s students	Medicine and Master’s/PhD students of other life and health sciences (different years; see Table 4, question 2)
Course size	16	12–25
Genetic testing strategy	Voluntary genotyping at the Life & Brain research Center, University Hospital Bonn, using the Illumina Infinium Global Screening Array (GSA) v3-MD	Voluntary genotyping (blood withdrawal) at the Institute of Human Genetics (TUM), molecular genetics laboratory, using the Illumina Infinium Global Screening Array (GSA) 24 v1.0
Course design	Four theoretical sessions of 3 h within two months, followed by a five-day block course with alternating theoretical and practice sessions including discussion rounds. Only a small fraction of personal genetic data was provided during the block course, raw data upon request after the course	3–4 full days block course with alternating theoretical and practical sessions including discussion rounds. Only a small fraction of personal genetic data was provided during the block course, raw data upon request after the course
Course content	Fundamentals of human genetics and molecular biology Clinical value and ethical implications of genetic testing Computational analysis of large-scale and individual genotyping data	Gain knowledge on interpreting and analyzing genetic variants based on public databases Gain knowledge to understand direct-to-consumer genetic testing options and the associated benefits and risks Fundamentals of human genetics and molecular biology Clinical value and ethical implications of genetic testing Direct-to-consumer-testing products Ancestry analyses Interpretation of genetic variants
Pedagogical approaches	Frontal education Computational exercises using the openSNP dataset to determine genetic ancestry and calculate a simple genome-wide association study and polygenic risk scores Small individual projects and presentation on genetic variants and disorders Panel discussion Multiple-choice exam	Frontal education Computational exercises to determine genetic ancestry using the openSNP dataset Guided analyses of genetic variants Student presentations on individual genetic variants and genetic disorders Discussion rounds Role play (simulated genetic counseling and adoption of different roles for discussion rounds)
Distinguishing features	Focus on computational analysis of array data using PLINK and additional resources	Focus on direct-to-consumer testing, interpretation of genetic variants, communication of genetic results to patients, and ethical aspects

The courses covered introductions to genetics and molecular biology, genetic epidemiology, and statistics, pharmacogenomics, medical genomics, and ethical aspects of genetic testing. The weighting of the individual topics was adjusted according to the students’ prior knowledge. Both courses covered the topics of variant annotation, interpretation, and risk estimation for individuals and costumers of DTC testing. The students were assigned small projects and presentation tasks to each student, accompanied by discussion rounds on specific genetic variants, their annotation, and associated risk.

A strong focus of AYPG was computational data analysis using a publicly available data set, covering standard computational tools and analyses in genetics (e.g., genome-wide association analysis and polygenic risk scores). Using the openSNP dataset, students learned how to read and filter genotyping data, perform quality control and basic genome-wide association studies, and calculate polygenic risk score using the PLINK tool [31]. Further discussion on the risks and benefits regarding genetic testing and a direct question and answer session with human geneticists were designed to stimulate a more sophisticated attitude and reflection on the topic.

In the GM course, on the other hand, the two major learning objectives were to gain knowledge on interpreting and analyzing genetic variants based on public databases and to understand options for DTC genetic testing and the associated benefits and risks. Accordingly, more emphasis was placed on interpreting DTC test results. Future physicians might be confronted with patients who have purchased DTC products. Thus, a critical understanding of the companies’ products and their benefits and risks is beneficial to medicine students. The analysis of genetic variants of individual patients was discussed in more depth during the TUM course, which is relevant for the clinical setting.

Regarding the students’ motivation to participate, only few differences between the two courses became apparent (Table 3): compared to TUM students, HPI students were significantly more interested in pharmacogenomics (*p* = 0.002) and showed a tendency for an increased interest in receiving or analyzing their own genomic data (*p* = 0.07). Overall, the highest motivation to participate in the courses was the general interest in genomics or genomic analyses (92% at HPI, 91% at TUM).

**Table 3:** Comparison of motivation to participate in the course between HPI and TUM

Topic	HPI (n = 26)	TUM (n = 55)	P-value	Effect size (V)
General interest in genomics/genomic analyses	92% (24)	91% (50)	1	0 (small)
To receive/analyze my own genomic data	85% (22)	64% (35)	0.07	0.19 (small to medium)
Interest in pharmacogenomics (e.g., the impact of genomic variants on medication effects)	77% (20)	38% (21)	0.002	0.34 (medium to large)
To gain knowledge about genomics/genomic analyses to apply it in my professional career	69% (18)	62% (29)^a^	0.61	0.05 (small)
Interest in research topics like genome-wide association studies	58% (15)	45% (25)	0.35	0.09 (small)
To learn about tools for variant interpretation and analysis	54% (14)	64% (35)	0.47	0.07 (small)
Interest in ethical issues in the context of genomic analyses	50% (13)	53% (29)	1	0 (small)
Interest in legal foundations of genomic analyses	50% (13)	44% (24)	0.64	0.03 (small)
Interest in ancestry analysis	46% (12)	47% (26)	1	0 (small)
To receive the credit points	42% (11)	38% (3)^b^	1	0 (small)
Interest in commercial genomic testing (“direct-to-consumer testing”)	38% (10)	45% (25)	0.63	0.04 (small)
To better understand the situation of patients when undergoing genomic testing	38% (10)	45% (25)	0.63	0.04 (small)

The p-values and effect sizes relate to the difference between answers provided by HPI and TUM participants. The information was assessed using HPI questionnaire Q4 2020 (n = 10), HPI questionnaire Q1 2021 (n = 16), TUM questionnaire Q4’ (n = 47), and TUM questionnaire Q1 2020/21 (n = 8). Q1 (pre-course) questionnaires were used where possible; questionnaire HPI Q4 2020 was used instead of Q1, as questions regarding the motivation to participate in the course differed considerably between the long- and short-version questionnaires. TUM Q4' was used, as this was the only questionnaire conducted for TUM courses taking place before the winter term 2020/21

^a^This question was included for TUM only in Q4’ (n = 47) and later included again for HPI questionnaires; it is missing in TUM Q1 2021/22

^b^Before the summer term 2021/22, students participating in GM did not receive credit points, therefore the question was only included for TUM Q1 2021/22 (n = 8) when the format was changed from a facultative to a compulsory elective course included in the curriculum

### Students find the genotyping component useful

Retrospectively, most students felt that genotyping in the course was useful for the learning experience (HPI: 89% [17 of 19], TUM: 92% [49 of 53]); 9 students who indicated that genotyping was a useful learning experience did not participate in the PGT component (Table 4). Almost all students would recommend offering PGT again in future courses (HPI: 95% [18 of 19], TUM: 98% [52 of 53]). 64% (34 of 53) of TUM participants expressed the opinion that a similar course should be an obligatory component of the medicine curriculum at universities.

**Table 4:** Combined results from retrospective questionnaires

Question	HPI (N = 19)	TUM (N = 53)	P-value	Effect size (V)
1. In which year did you participate in the course? (ST = summer term, WT = winter term)	ST 2020: 10 ST 2021: 9	ST 2017: 5 WT 2017/18: 12 ST 2018: 15 WT 2018/19: 6 WT 2019/20: 9 WT 2020/21: 6	–	–
2. Did you study medicine?^f^	Yes: 4 No: 15	Yes: 36 No: 11	–	–
3. Did you participate in personal genotyping as part of the course?	Yes: 17 No: 2	Yes: 44 No: 9	0.72	0.04 (small)
4. From today’s perspective, would you have yourself genotyped again?^b,a[3=yes]^	Yes: 6 No: 1	Yes: 6 No: 0	1	0.35 (large)
5. From today’s perspective, would you have yourself genotyped?^b,a[3=no]^	Yes: 1 No: 1	–	–	–
6. Did you collect the password for your complete genotype data? ^*a[3*=*yes]*^	Yes: 13 No: 4	Yes: 35 No: 9	1	0 (small)
7. Do you still plan to collect the password for your complete genomic data?^c,a[6=no]^	Yes: 3 No: 1	–	–	–
8. Did you conduct analyses with your own data beyond the course?^e,a[6=yes]^	Yes: 7 No: 6	Yes: 14 No: 21	0.52	0.08 (small)
Genetic ancestry^c^	Yes: 2 No: 11	Yes: 2 No: 4	0.56	0.07 (small)
Pharmacogenomics^c^	Yes: 2 No: 11	Yes: 4 No: 2	0.05	0.39 (medium to large)
Wellness traits^c^	Yes: 4 No: 9	Yes: 1 No: 5	1	0.02 (small)
Carrier status^c^	Yes: 1 No: 12	Yes: 2 No: 4	0.22	0.17 (small to medium)
Polygenic diseases^c^	Yes: 0 No: 13	Yes: 2 No: 4	0.09	0.32 (medium to large)
No, I just wanted to receive my data^c^	Yes: 6 No: 7	Yes: 2 No: 4	1	0.01 (small)
9. Do you plan to conduct further analyses with your own genomic data?^a[6=yes or 7=yes]^	Yes: 14 No: 2	Yes: 22 No: 13	0.1	0.2 (small to medium)
10. Has this course changed your attitude towards personal genotype analysis?	Yes: 15 No: 2 I don’t know: 2	Yes: 25 No: 24 I don’t know: 4	0.02	0.32 (medium to large)
11. How did the course change your attitude?^a[10=yes]^	More positive: 4 More critical: 11	More positive: 7 More critical: 18	1	0 (small)
12. Would you have participated in personal genotyping as part of the course if it had been offered via a non-European private company, such as 23andMe?	Yes: 7 No: 12	Yes: 11 No: 42	0.22	0.13 (small to medium)
13. Do you think that personal genotyping in the course context is useful for the learning experience?	Yes: 17 No: 2	Yes: 49 No: 4	0.65	0 (small)
14. Would you recommend to offer personal genotyping in future courses again?	Yes: 18 No: 1	Yes: 52 No: 1	0.46	0 (small)
15. How important do you consider the treatment of ethical aspects in the AYPG course?	Absolutely necessary: 12 Rather important: 6 Neutral: 1 Rather unimportant: 0 Completely unimportant: 0	Absolutely necessary: 40 Rather important: 8 Neutral: 5 Rather unimportant: 0 Completely unimportant: 0	0.3	0.19 (small to medium)
16. Do you feel adequately trained to analyze and interpret genomic data beyond the course?^c^	Yes: 8 No: 11	Yes: 2 No: 4	1	0 (small)
17. Would you recommend personal genotyping to relatives or friends who have not taken the course?	Yes: 13 No: 6	Yes: 16 No: 37	0.01	0.31 (medium to large)
18. Should personal genotyping be allowed without genetic counseling by a medical professional?	Yes: 4 No: 15	Yes: 16 No: 37	0.56	0.05 (small)
19. Do you think that a similar course should be obligatory in medical studies at universities?^d,a[2=yes]^	–	Yes: 34 No: 19	–	–

The p-values and effect sizes relate to differences between responses by HPI and TUM individuals. The information was assessed using HPI questionnaire Q4 2020 (n = 10), HPI questionnaire Q4 2021 (n = 9), TUM questionnaire Q4’ (n = 47), and TUM questionnaire Q4 2020/21 (n = 6)

^a[previous question=answer]^Whether question was asked depends on the answer to previous questions

^b^Question was not asked in Q4’ and HPI Q4 2020

^c^Question was not asked in Q4’

^d^Question was only asked at TUM

^e^Yes/no question in Q4’ was combined with more detailed answers from other questionnaires: if any analysis was selected by a participant, the answer was counted as “Yes”

^f^Was not explicitly asked in TUM questionnaires. Was deferred from more detailed question in Q4’ “What did you study or which education did you have when you participated in the course?”—the answer “Medicine” was counted as “yes”, answers “Genetic and Genomic Counseling” (N = 4) and “Other educational background” (N = 7) as “no”. In TUM Q4 2020/21, all students were medical students

The majority of questionnaire respondents participated in PGT (HPI: 89% [17 of 19], TUM: 83% [44 of 53]). Many students also collected the passwords to access their full genetic data (HPI: 76% [13 of 17], TUM: 66% [35 of 53]) or still planned to collect it at the time of the questionnaire (HPI: 3 of 4). Overall, less than half of the questionnaire participants who collected their password had conducted their own analyses with their full data at the time of Q4 (HPI: 54% [7 of 13], TUM: 40% (14 of 35)). Of all students who did or still planned to collect their passwords, most still intended conduct further analyses (HPI: 88% [14 of 16], TUM: 63% [22 of 35]).

Of all HPI and TUM Q4 2020/21 survey participants who collected their passwords (N = 19), 6 analyzed pharmacogenomic markers (HPI: 2 of 11, TUM: 4 of 6), 5 wellness traits (HPI: 4 of 11, TUM: 1 of 6), 4 genetic ancestry (HPI: 2 of 11, TUM: 2 of 6), 3 carrier statuses (HPI: 1 of 11, TUM: 2 of 6), and 2 their risk for polygenic diseases (HPI: 0 of 11, TUM: 2 of 6). Notably, only 40% (10 of 25) of students felt adequately trained to interpret genomic data outside of the course (HPI: 42% [8 of 19], TUM: 33% [2 of 6]).

Most students stated retrospectively that they would not have participated in the PGT component if it had been conducted by a non-European private company such as 23andMe: 63% (12 of 19) of HPI students and 79% (42 of 53) of TUM students. In responses to open-ended questions at the AYPG course in 2020 regarding whether the students would participate in PGT (if it was allowed in Germany), 9 stated that they would not have been able to afford WGS-based PGT. Furthermore, 10 students voiced concerns regarding data security and handling and would make the decision contingent on which institution provided the WGS and how this institution handled the data. Finally, the majority of HPI and TUM participants felt that personal genotyping should not be allowed without prior genetic counseling (HPI: 79% [15 of 19], TUM: 70% [37 of 53]). Interestingly, the two groups of students had different opinions about whether they would recommend PGT to relatives and friends who have not taken the course: 30% (16 of 53) of TUM students would recommend it, compared to 68% (13 of 19) of HPI students (*p* = 0.01).

### Students changed their attitude towards genotyping throughout the course

The course changed the attitudes of many—especially HPI—students towards personal genotype analyses (HPI: 79% [15 of 19], TUM: 47% [25 of 53]; *p* = 0.02), with students typically becoming “more critical” (HPI: 73% [11 of 15], TUM: 72% [18 of 25]).

This can partially be confirmed by following the responses across the questionnaires (Table 4 and Additional file 5). During the course conducted in 2020, HPI students showed a trend of becoming more skeptical regarding the interpretability and usefulness of genetic data. Specifically, students mostly agreed that PGT results are not predictive (average values reported for each time point after grouping the values to agreement = 1, neutral response = 0, and disagreement = − 1; time point Q1: 0 (neutral), Q2: 0.57 (agree), Q3: 0.75 (agree); *p* for Q1 vs. Q3 = 0.0477; question not asked in Q4). They eventually rather disagreed that PGT is useful to inform family members about health risks (Q1–Q3) and that they would recommend PGT to relatives and friends (Q4) (time point Q1: 0.67 (agree), Q2: 0.83 (agree), Q3: − 0.5 (disagree), Q4: 0.33 (rather agree); *p* for Q2 vs. Q3 = 0.053). The students also eventually rather disagreed that PGT is an opportunity to obtain information that can help them improve their health and wellbeing (time point Q1: 0.62 (agree), Q2: 0.75 (agree), Q3: − 0.12 (rather disagree); *p* for Q2 vs. Q3 = 0.053; question not asked in Q4). In addition, HPI students disagreed during the entire course that genetic testing should be allowed without counselling (time point Q1: − 0.5 (disagree), Q2: − 0.5 (disagree), Q3: − 1 (disagree), Q4: − 0.67 (disagree); *p* for Q2 vs. Q3 = 0.149) and were more likely to believe that they could learn unwanted information from PGT (time point Q1: − 0.62 (disagree), Q2: − 0.25 (rather disagree), Q3: 0 (neutral); *p* for Q1 vs. Q3 = 0.088; question not asked in Q4). Consistent with the fact that fewer TUM students reported an attitude change, their responses did not change as much over the course as they did for HPI students, although HPI responses changed less in 2021 (mean absolute change of average responses HPI 2020: 0.71; TUM 2020/21: 0.32; HPI 2021: 0.48). In both groups, most students had not yet conducted the genetic analyses they had initially planned at the time of Q3 or Q4.

In open-ended questions at the AYPG course 2020, students provided information regarding the analyses they planned to carry out and their reasons. In Q1, students stated overall curiosity (n = 6) and improvement of analysis skills relevant for their future careers (n = 3) as reasons for further analyses. In Q2, the students stated that they were interested in finding out more about their healthcare risks (n = 4) and to improve their skillset (n = 3). In Q3 (after the course), some students expected to require further support for being able to properly understand their data (n = 2) or were concerned about learning unwanted information (n = 3). Others specifically did not want to further investigate risk for serious healthcare conditions but instead planned to analyze their genetic ancestry (n = 2) or acquire pharmacogenomic information (n = 2).

## Discussion

In the USA, PGT was already implemented as an educational method for university students in several, mostly medical and pharmaceutical schools. By contrast, at European universities, the genomics courses for DH students at the HPI Potsdam and for medical students at the TU Munich are, to the best of our knowledge, the only courses that implemented genetic testing with genome-wide coverage at the time when we collected our questionnaire data. The Medical University of Innsbruck has established a PGT course in Genomic Medicine, modelled on the GM course at TUM as part of their Master’s program in Genetic and Genomic Counselling in 2022.

While the course content differed between the courses at HPI and TUM, the implementation of PGT within the context of the courses was conducted in a similar manner. The use of PGT as part of the offered courses lies outside of the legal restriction of the GenDG, as it is only intended for research and educational purposes. At both Universities, ethical boards explicitly approved the courses and genetic testing for students, performed by a research institution without monetary gain. Students preferred this test setup to the possible alternative of conducting DTC testing via non-European private companies. The implementation described here could serve as a blueprint for universities in Europe when establishing a similar concept for genomics courses at their site.

The concept of personal genotyping for educational purposes can be beneficial for genomic courses of different study programs, as described for the AYPG course at HPI and the GM course at TUM. The idea of adding this educational component to a genomics course was motivated by the assumption that the students’ engagement and learning would be increased during the course and that personal reflection would make them more sensitive to genetic testing in general. Overall, the course questionnaires confirmed that students perceived the PGT component as useful and motivating. The vast majority of students would recommend keeping the PGT component as part of the course and advised to add similar courses to the curriculum of other universities. Similarly, medical, PharmD and undergraduate students from several US universities described PGT in the context of their genomic education as an important part of their curriculum [15, 20, 21] and as useful for their future practice [19, 32].

Our data did not allow for a comparison regarding whether knowledge gain differed between genotyped and non-genotyped students because of the high participation rates in the PGT and small total sample sizes. Nevertheless, both higher test scores and self-reported knowledge, as well as a higher engagement in the course by students participating in PGT were previously reported [15, 17, 21]. However, the benefits must outweigh any risks associated with the genetic testing, and risks must be minimized to justify the implementation of such educational techniques.

In the university context, these risks include anonymity, confidentiality, and coercion [11, 14, 33]. The course itself is an elective part of the study program, and PGT was not a requirement for course participation. An honest broker system made sure that the genetic data and corresponding personal information were strictly separated. The costs of genetic testing were covered by the Life & Brain Research Center (HPI) and the Institute of Human Genetics (TUM) so that students could undergo PGT regardless of their financial situation. Additionally, genetic testing was not conducted through a private company to mitigate the risk of conflicts of interests and of privacy issues including data leakage.

Furthermore, a major risk of genetic testing in general is that unwanted information may be learned. For the two courses in Potsdam and Munich, just a fraction of low-risk personal genetic information was returned to the students during the course. Only after completion of the course, students could request their complete data, minimizing the risk of making an uninformed decision to explore personal genetic information. At different stages of the courses, risks and benefits of genetic testing were discussed, so that continuous learning of the students regarding this topic was assured.

Most students who participated in the PGT component of the course requested or planned to request the password to decrypt their genetic data, but primarily non-disease related analyses had been conducted at the time of Q4 and Q4’. Many students did not conduct subsequent analyses with their data at all. The questionnaire responses also indicate a change in attitude towards genetic testing among HPI and TUM students: Most students answered that they had become more critical (as opposed developing a more positive attitude). However, it is not explicitly clear how the students interpreted the predefined answers “more critical” and “more positive”, available for the question on how students changed their attitude. These ambiguous answering options constitute a limitation of our study; in future questionnaires, either the wording should be more precise or further follow-up questions should be included. However, based on the overall trend of the students’ responses over the course and given our personal interactions with the students during the courses, e.g., in discussions rounds, we feel confident that the response “more critical” can typically be interpreted as “more reflective/aware of ethical implications”. We presume that this trend reflects the intense discussions of ethical aspects conducted throughout the courses (Table 1, Additional file 6). As shown with the example of the AYPG and GM courses, the content and structure of genomic courses should be tailored to the specific student population. One important aspect is that the desired learning outcomes differed between medical and DH students. While the study program in Medicine requires a stronger focus on patient-doctor interactions and patient-oriented interpretation of results, DH students were more intensely trained in computational analyses of larger genomic datasets.

Questionnaire results showed that the different focus areas were consistent with the students’ motivation to participate in the course. However, the interest of students may have been modified by the preceding contents and lectures provided at HPI and TUM. For instance, the high interest in pharmacogenomics within the HPI student cohort can be explained by a preceding lecture covering basic concepts of pharmacogenomics that all students from the course were required to attend. This heterogeneity in the student populations constitutes a limitation for our comparisons of the survey data across the two institutions.

There are further limitations related to the course questionnaires: One major limitation is the small sample size, which is why the results were not further stratified by educational background (and for Q4’ by course years). This may have introduced biases. Moreover, the voluntary survey participants could represent a subset of more positive and motivated or more critical students. While the survey results can certainly provide insights and qualitative trends, no clear conclusions can be drawn from the quantitative analyses. Furthermore, the re-design of the survey after the first HPI iterations limits the comparability of the responses between all iterations of the questionnaires. Nonetheless, we consider our approach a valuable evaluation of the course concept.

Finally, understanding genomic information is important not only in the context of the DH and Medicine study programs, but also for the general population, especially regarding personalized health services that provide genetic information to their customers. The majority of students from HPI and TUM supported the idea that genetic counseling should be mandatory when personal genotyping is conducted, as is required by law in Germany. This response is to some degree surprising, as the students received their own raw genetic data without further counseling and at least the HPI students would recommend PGT to family and friends who did not even take the course.

These results are in line with the observations made by Weitzel et al.: Even though most students participating in PGT of selected pharmacogenomic SNPs agreed that genotyping was useful for the course and improved their understanding of the content, the number of students considering recommending DTC to patients decreased over the course. As a possible reason, the authors suggested the potential increase in understanding the complexity and therefore the demand for professional support in interpreting results [20].

## Conclusion

The application of voluntary PGT in two genomic courses at HPI and TUM was perceived as useful by most students across study programs and demonstrates the feasibility of the implementation of an innovative course design at German universities. Further investigation including higher sample sizes are required for more robust quantitative analyses.

## Supplementary Information

**Additional file 1**. AYPG course information sheet with consent form and appendix containing list of traits that can be assessed from partial data.

**Additional file 2.** List of variants that were returned to students during the AYPG course.

**Additional file 3.** Single PDF questionnaires.

**Additional file 4.** All conducted questionnaires with survey periods and number of participants.

**Additional file 5.** Comparison of questionnaire results between different questionnaires in one course year (*p*-values < 0.1).

**Additional file 6.** Schedule of the Genomic Medicine course at TUM (winter term 2020/21).

**Additional file 7.** Aggregated questionnaire response data.

## Data Availability

The datasets generated and analyzed during the current study are not publicly available due to data privacy restrictions but are available from T.S. (tamara.slosarek@hpi.de) on reasonable request. The questionnaire responses are available in aggregated form, with pseudonyms and open text responses removed (Additional file 7).
