# Supplementary material for: Implementation and evaluation of personal genetic testing as part of genomics analysis courses in German universities
Source: BMC Med Genomics. 2023 Apr 5;16:73. doi: 10.1186/s12920-023-01503-0 (PMC10074719; doi:10.1186/s12920-023-01503-0)
Supplement: Supplementary file 1 — Additional file 1. AYPG course information sheet with consent form and appendix containing list of traits that can be assessed from partial data. [file 12920_2023_1503_MOESM1_ESM.pdf]

Supplement 1

AYPG course information sheet with consent form and appendix containing list of traits that can be assessed from partial data.

Analyze Your Personal Genome

- INFORMATION FOR COURSE PARTICIPANTS -

Dear student,

You have decided to participate in the course *ANALYZE YOUR PERSONAL GENOME* (AYPG). The course is offered by Hasso Plattner Institute (HPI) Digital Health Center (DHC), Chair (Fachbereich) – Digital Health and Personalized Medicine. The basis for the course is genome-wide genotyping data, obtained prior to course. Two options are available: With option A, you will receive an already existing anonymized genome-wide genotype file for analysis. For option B your own genotype data will be determined and then analyzed by yourself. Regardless of which option you choose, we ask you to read the following information carefully. Please discuss all outstanding questions with the course instructors prior to the start of the course.

1. What is the course about?

The purpose of the course "Analyze Your Personal Genome" is to educate students about the ethical, biological and mathematical aspects of using personal genome information in health care, medical practice and biomedical research.

We will focus on practical issues using public available individual genomic data and other genomic data at population level to learn about the basic concepts of statistical genomics. Students will have the opportunity to explore a selection of their own genomic data to understand the predictive character of genomic variants and their significance for diagnosis, prognosis and therapy in medicine.

The content of the course covers genotyping techniques, bioinformatics and statistical basics including the linking of the collected data with public available databases and the interpretation of sequence variation. We will discuss scientific issues such as determining the genetic lineage of individuals, learning from genomes about the migration and evolution of the human population, and how natural selection has shaped human genomes. Furthermore, the course will discuss medical aspects: how to detect disease-causing genetic mutations, how to predict diseases such as diabetes based on genomic data, and how genetic information can be used to recommend clinical treatments. Additionally, we will talk about direct-to-consumer tests commonly used to inform about non-medical, general wellness traits (such as athletic ability, hair color, earwax type, wine taste preferences, -for example), or help individuals explore their genetic ancestry. Practical exercises will form the core of the course. In addition, the legal and ethical dimension of genomic medicine will be discussed in discussion groups.

Note: All procedures of this course, including genetic counseling, genotyping, etc., are being done for educational purposes only.

The genotyping used for the course involves selected variants, so called single nucleotide variants (SNVs, n= 730,059) distributed throughout the genome and will be carried out using

the Illumina Infinium Global Screening Array (GSA v3-MD). The genotyped SNVs can be divided into two groups: Group I consist of variants distributed relatively evenly across the genome and are used in genome-wide association studies for research purposes (so-called backbone SNVs, n~92%). Group II contains selected variants with potential clinical relevance (so-called clinical research SNVs). Both groups contain coding variants with the potential to alter protein sequences (~7% in total). During the course only SNVs from group I are accessible to the course participants - SNVs not suitable for determining the individual risk of rare monogenic diseases and limited to determining the risk of common multifactorial diseases. Additionally, a pre-selected subset of possible SNVs from group II are available for course participants: Only the carrier status of recessive diseases can be determined via these SNVs (Appendix 1). In addition, SNVs that play a role in the expression of twelve different characteristics are made available for analysis (Appendix 2), as well as SNVs in genes that are associated with the effect of twelve different drugs (Appendix 3). It is therefore possible that the genome-wide genetic analysis described above may lead to the detection of the carrier of recessive diseases, the detection of selected traits such as lactose intolerance or the detection of altered sensitivity to drugs.

2. Optional pre-genotyping genetic counseling

You can discuss whether to obtain your own personal genotyping data with a healthcare professional during the introductory course.

3. What types of information can be learned from genotyping and what are the limitations?

To minimize your “risk” of learning unwanted information during this course, we will limit your access to SNVs belonging to the genotyping backbone (group I) and a subset of the clinical genome (group II) as describes above.

In general, and especially also considering whole genome sequencing, the following types of information can be learned from genotyping:

1. It is expected that you will find a large number of unique SNVs in your DNA that may be difficult to interpret at the present time;
2. Your personal genomic data is being provided to you for informational, educational and research purposes only, and are not intended to substitute for a medical diagnosis;
3. The amount of information that will be generated from genotyping will be vast and may be of unclear significance to your health;
4. Our knowledge about the meaning of variations in the genome is growing rapidly but is largely incomplete;
5. Potentially important SNVs that may be important to your health may be masked;
6. Potentially important SNVs that may be important to your health may be missed;
7. Some SNVs may be identified that are likely to be of clinical significance;
8. Some of this information can be disturbing;
9. It is very important for you to carefully think about whether you wish to analyze your own genome data through which many different types of personal information may arise.

We would like to remind you that the types of variants that you might find in your DNA include (but are not limited to):

1. Variants related to ancestry;
2. Variants in genes that affect how your body uses certain medications (pharmacogenomics);
3. Variants in genes that might increase or decrease your risk of developing conditions like heart disease and obesity;
4. Variants in genes that might increase or decrease your risk of developing certain types of cancers;
5. Variants in genes that might increase or decrease your risk of developing psychiatric disorders, such as schizophrenia, and neurologic disorders, such as Alzheimer's disease;
6. Variants that indicate that you are a carrier for a disease gene (i.e. that you have one "disease" copy of a gene, and one healthy copy of the same gene, that while a carrier does not have the disease, there is a chance that family members might either have the disease or also be carriers, and that if you do find that you are a carrier of a disease gene, you may wish to review whether any family members might be at risk, and if so, how to approach them to discuss testing);
7. Variants that indicate that you have a treatable disease that you were not aware of (if you find that you have a disease that is treatable, you may wish to be referred for appropriate confirmatory testing and treatment);
8. Variants that indicate that you are likely to develop a serious, potentially fatal disease, for which there is no treatment or cure.

It is also possible that no information of any clinical significance will be revealed.

Please note that this is an academic class and that the genotyping is not being performed under a clinical protocol. No results can or should be used directly in clinical decision-making. During the course you will have the opportunity to engage a genetic counselor and human geneticists to follow-up on any questions regarding potentially clinically significant findings.

You are encouraged to seriously consider what information you would like to know from genotyping and what you would prefer not to learn about. It may not be possible for you to avoid learning certain types of information about yourself, and if you feel very strongly that you do not want to learn some types of information that could arise, then you should consider using a publicly available, anonymous genome rather than your own genomic data.

You will have the opportunity to change your decision at any time, including after you actually receive your personal genomic data.

You are eligible to participate in the course whether you prefer to analyze your own or an anonymous genome.

To reiterate, you do not have to obtain your personal genome data, in order to participate in the course.

4. What do you do?

If you decide on option A (assignment of an anonymous data set), you will analyze an anonymous control data set in the course. If you decide on option B (individual typing), we need a DNA sample from you. DNA will be ascertained in form of a saliva sample (~2 ml saliva) using DNA Genotek's saliva-based DNA collection kits (OG-500). As soon as your saliva sample is taken, the tubes will be labeled with a sticker that contains a unique "barcode". No other identifying information will be on the saliva specimens. Another sticker with the same barcode will be placed on the back of your genotyping information sheet and immediately put in a locked cabinet. The link between your barcode number and your identity will be kept confidential and will only be known to the designated course "honest broker". You will keep a third sticker with your own barcode and it will be your responsibility to keep the barcode confidential.

5. How are the samples being processed and how are the data and samples protected?

Saliva specimens are being collected at DHC in Potsdam. We will obtain genetic material (DNA) from the saliva cells at Life & Brain Research Centre, University Hospital of Bonn. Your sample will only be identified by the unique barcode. No personal information (such as name, date of birth) will be provided to the laboratory that analyzes your sample or provides the initial annotation of the data.

Please note that the genotyping test is being done for educational purposes only.

The raw data files (i.e. raw intensity data files) produced by the genotyping platform will be shared via SFTP along with a set of control samples. Using Illumina's GenomeStudio v2.0 software, genotype calling, and quality control of the data will be carried out according to standard operating procedures using the anonymized data. After genotyping is complete, DNA samples will be destroyed and anonymized files containing the genotypes (VCF or PLINK files) with filenames containing only the anonymous barcodes described above will be shared with the respective students. Only you know which data set belongs to you using your individual barcode. To guarantee data privacy, you must not share your personal barcode with others. If you chose to share your barcode with others (e.g. other course participants or course teaching staff), those people might be able to link your genetic data to you. It is your responsibility to keep your barcode confidential.

If you elect to use an anonymous genome, you will be matched to the most similar (in terms of sex and ethnicity) available genome. These relevant files will use the same naming scheme as those described above so as to be as indistinguishable as possible from those genotyped for the course. The honest broker will share the information on name and location of these files to you.

For the practical part of the course you will be granted access to a private and secure virtual machine on the DHC lab – to which only you have permission to access.

The genotyping data will not be retained by the institution or used for any purpose other than to provide to you for educational purposes and will be deleted after the course is finished.

Course participants can receive their own raw genotype data set after completion of the course and on their own responsibility upon request.

6. What are my responsibilities?

If you decide to take part in the genotyping component of the course, you will be responsible for the following things: Attending the information session and completing the saliva draw. Furthermore, it is your own responsibility to keep your barcode – the re-identification tag – confidential.

7. Are there costs or payments that may result from participation?

If you agree to take part in the genotyping component of the course, you will not be paid for doing so. The genetic counseling sessions and genotyping will be provided to you at no cost.

8. What are the benefits of this course?

The presentation and interpretation of genomic variation is fundamental for modern medicine. Genetic variants determine disease entities, provide causal relationships in diagnostics, have a preventive character and serve as target structures for drugs. The term "genomic medicine" reflects the increasing importance of individual genetic variants in diagnosis, prevention and therapy of diseases. Through the analysis of genomic data and in particular, by analyzing your own genomic data, you gain an immediate and lasting impression of the practice of molecular genetic diagnostics and research. We cannot and do not guarantee or promise that you will receive any direct benefit from participating in the genotyping component of this course. However, others might potentially benefit from your participation in this component of this course, in particular if you decide to contribute to the course evaluation helping us to improve the course in the future and understand expectations, wishes, needs and concerns of course participants. Results of the course-accompanying evaluation might be part of a scientific research paper.

9. What is the risk?

There are risks, discomforts, and inconveniences associated with any procedure of this kind. These deserve careful thought. In addition to what is described below, there may be unforeseeable risks that occur as a result of genotyping and your interpretation of the data this produces.

Risks related to coercion

One risk of participating in the class is that you will feel coerced into getting your genetic data in order to please the course directors and may feel that if you don't do these things then you may jeopardize your grades.

You absolutely do not have to get your genome genotyped. Your genome data will be kept completely separate from your name. Your genome data will be accessible only to you. The course directors will never see your genome data, nor know whether or not you chose to receive your own genome data or that of an anonymous, publicly available genome. In addition, the personal genotyping component of the course will be excluded from grading.

Grades will only be allocated to exercises using publicly available data, and the final exam aimed at testing your basic understanding of the course content.

Risks related to saliva-sample based DNA collection

Risk of complications such as hemorrhage or infection, as connected with blood-sample based DNA collection, is non-existent for saliva-based DNA collection.

Risks related to learning genetic information

There is a chance that you may learn that you carry a gene mutation that may increase risk for a specific medical condition. Although you will have the option of seeking a referral for medical help or risk management as appropriate this knowledge might be upsetting and may cause anxiety or psychological distress. As described above, some of the conditions you learn about may have treatment or screening options available, while others may not. Some of these conditions may also be potentially stigmatizing. Please think about if you want this information before the data is available. You may learn that a family member is at risk of developing certain medical conditions or diseases. You may also learn that ancestry or parentage is different than previously thought. This may cause some psychological distress and can be upsetting.

Risks related to privacy

Your privacy is very important to us, and we will use many safety measures to protect it. However even with all of these protections, there is the possibility that the genotyping data derived may, even when presented without other identifying factors, allow you to be re-identified, and therefore we cannot promise anonymity, particularly if you choose to publish or share your genome data with others.

Specific illnesses and known genetic problems may be found by examining DNA. In the future, insurance companies may use this information to determine if someone is able to be insured by their company. If you tell your family doctor that you have had your genome genotyped, or if you tell your doctor about any specific aspects relating to your genotyping data, this information may then become part of your medical record with this doctor. Insurance companies routinely have access to such records. You will be the sole owner of your genome data, and so there is no risk of your information being released about you or your family to your doctor unless you choose to do so. While your DNA is being genotyped, the investigators will keep your identifying information separate from the DNA. The DNA will be linked to your personal information only through an ID number – barcode. We will keep linking information under lock and key and confidential to the extent permitted by law.

The course personnel will be able to see your anonymized genomic data and will perform quality control and filtering of variants, however, they will not be able to link the genotyping files with your name. Additionally, the files containing your genotyping data will be stored at one location together with files from other course participants and additional control data sets to minimize risk of de-identification.

The course personnel is trained in compliance with the data protection regulations (DSGVO).

There is a small risk that you may face discrimination on the basis of genetic predispositions that are identified through your genome being genotyped. Sometimes patients have been required to furnish information from genetic testing for health insurance, life insurance, and/or a job. The Gendiagnostikgesetz of 2009 (GenDG), generally makes it illegal for health

insurance companies, and employers to discriminate against you based on your genetic information.

There always exists the potential for loss of private information; however, there are procedures in place to minimize this risk.

10. Is it possible to withdraw from the analysis of your own genotype data?

You may decide not to have your genome genotyped without any penalty. This choice is totally up to you. Course participants can also waive option B (analysis of own data) during the course and are then assigned a control data record (option A). You may stop taking part in the genotyping component of the course at any time without any penalty.

If you decide to stop being in the genotyping component of the course, please contact the course coordinator and honest broker.

11. Withdrawal without your consent

The sponsor or the institution may stop your involvement in the genotyping component of this course at any time without your consent. This may be because the course is being stopped, the instructions of the course staff have not been followed, the staff or investigators believe it is in your best interest, or for any other reason. If specimens or data have been stored as part of the project, they too can be destroyed without your consent.

If consent to store the tissue sample is withdrawn at any time prior to it being destroyed as per the project protocol, the repository storing the sample will promptly destroy the sample.

12. Contact person(s)

TBD

Analyze Your Personal Genome

- DECLARATION OF CONSENT –

FOR COURSE PARTICIPANT FOR THE GENOME-WIDE GENETIC ANALYSES WITHIN THE COURSE

Name: ______________________________

Address: ______________________________

Phone: ______________________________

Course doctor: ______________________________

I was informed about methods and risks in the course *ANALYZE YOUR PERSONAL GENOME*. I agree that

- I have a saliva sample being taken
- Genome-wide genetic analyses are performed on the samples with the GSA array

I have been informed and agree that the data collected for the course will be deleted after the end of the course. Third parties will not be allowed to view personal data.

For further information, the study team may need to contact the course participants again at a later date.

I would prefer to be informed via: O Email O Phone O Letter

Email: ______________________________

Address: ______________________________

Phone: ______________________________

I hereby declare my agreement to participate in the course with option B (collection and analysis of my own genotype data).

I have read the information sheet and I was informed orally about genotyping and the use of the data in the course. I had ample time to think about this decision. All questions about the course were answered. I know that the course doctor mentioned below will be available in the future, if I have questions about the course or my rights as a course participant. I have received a copy of the information sheet and the consent form.

On request, I can receive the raw genotype data collected during the course and can analyze it on my own responsibility and at my own risk beyond the course. The supervisors of the course are not responsible for analyses carried out outside the course. I am aware that from my own analysis of the genotype data I could possibly obtain information about

disease risks both from myself and from relatives. I am conducting these analyses on my own responsibility and will contact a human genetic counselling service if necessary.

……………………………..	………………………………………………………..
Place, Date	Signature of course participant
……………………………..	………………………………………………………..
Place, Date	Signature of informing course doctor

Contact details of the course doctor:

(Name, Address, Phone, Email)

Analyze Your Personal Genome

- APPENDIX -

1. Recessive diseases for which the carrier status can be determined

ARSACS
Agenesis of the Corpus Callosum with Peripheral Neuropathy (ACCPN)
Autosomal Recessive Polycystic Kidney Disease
Beta Thalassemia
Bloom's Syndrome
Canavan Disease
Congenital Disorder of Glycosylation Type 1a (PMM2-CDG)
Connexin 26-Related Sensorineural Hearing Loss
Cystic Fibrosis
D-Bifunctional Protein Deficiency
DPD Deficiency
Dihydrolipoamide Dehydrogenase Deficiency
Familial Dysautonomia
Familial Hyperinsulinism (ABCC8-related)
Familial Mediterranean Fever
Fanconi Anemia (FANCC-related)
G6PD Deficiency
GRACILE Syndrome
Gaucher Disease
Glycogen Storage Disease Type 1a
Glycogen Storage Disease Type 1b
Hereditary Fructose Intolerance
Junctional Epidermolysis Bullosa (LAMB3-related)
Leigh Syndrome, French Canadian Type (LSFC)
Limb-girdle Muscular Dystrophy
Maple Syrup Urine Disease Type 1B
Medium-Chain Acyl-CoA Dehydrogenase (MCAD) Deficiency
Mucolipidosis IV
Neuronal Ceroid Lipofuscinosis (*CLN5*-related)
Neuronal Ceroid Lipofuscinosis (*PPT1*-related)
Niemann-Pick Disease Type A
Nijmegen Breakage Syndrome
Pendred Syndrome
Primary Hyperoxaluria Type 2 (PH2)
Rhizomelic Chondrodysplasia Punctata Type 1 (RCDP1)
Salla Disease
Sickle Cell Anemia
Sjögren-Larsson syndrome
Tay-Sachs disease
Tyrosinemia Type I

Usher Syndrome Type I (*PCDH15*-related)
Usher Syndrome Type III
Zellweger Syndrome Spectrum

2. Characteristics whose value can be partially determined

Alcohol Flush Reaction
Bitter Taste Perception
Blond Hair
Earwax Type
Eye Color
Hair Curl
Lactose Intolerance
Male Pattern Baldness
Muscle Performance
Norovirus Resistance
Red Hair
Smoking Behavior

3. Effect of drugs that can be partially determines

Proton Pump Inhibitor (PPI) Metabolism (*CYP2C19*-related)
Warfarin (Coumadin®) Sensitivity
Phenytoin Sensitivity (Epilepsy Drug)
Sulfonylurea Metabolism
Thiopurine Methyltransferase Activity
Abacavir Hypersensitivity
Acetaldehyde Toxicity
Clopidogrel (Plavix®) Efficacy (*CYP2C19*-related)
Fluorouracil Toxicity
Hepatitis C Treatment Response
Pseudocholinesterase Deficiency (Mivacurium, Suxamethonium)
Simvastatin-Induced Myopathy
