# Supplementary material for: Implementation and evaluation of personal genetic testing as part of genomics analysis courses in German universities
Source: BMC Med Genomics. 2023 Apr 5;16:73. doi: 10.1186/s12920-023-01503-0 (PMC10074719; doi:10.1186/s12920-023-01503-0)
Supplement: Supplementary file 3 — Additional file 3. Single PDF questionnaires. [file 12920_2023_1503_MOESM3_ESM.pdf]

Supplement 3.
Single PDF questionnaires

Automatic English translations of German questionnaires were created with DeepL (www.deepl.com) and reviewed by the authors.

HPI 2020 Q1 ..... **2**

HPI 2020 Q2 ..... **25**

HPI 2020 Q3 ..... **45**

HPI 2020 Q4 ..... **66**

TUM Q4‘ **71**

TUM Q4‘ (AUTOMATIC ENGLISH TRANSLATION) **75**

TUM 2020/21 Q1 ..... **79**

TUM 2020/21 Q1 (AUTOMATIC ENGLISH TRANSLATION) **82**

TUM 2020/21 Q2 ..... **85**

TUM 2020/21 Q2 (AUTOMATIC ENGLISH TRANSLATION) **88**

TUM 2020/21 Q3 ..... **91**

TUM 2020/21 Q3 (AUTOMATIC ENGLISH TRANSLATION) **95**

TUM 2020/21 Q4 ..... **99**

TUM 2020/21 Q4 (AUTOMATIC ENGLISH TRANSLATION) **104**

HPI 2021 Q1 ..... **109**

HPI 2021 Q2 ..... **113**

HPI 2021 Q3 ..... **116**

HPI 2021 Q4 ..... **121**

HPI 2020 Q1

Page 01
Intro

Questionnaire 1: Pre-Course

Analyse Your Personal Genome (AYPG)

Dear AYPG student,

The purpose of this questionnaire is to learn about your expectations of the course and your opinion regarding analyzing your own genome and genome analysis in general. In total, we will ask you to answer three consecutive questionnaires at different time points in the course.

This is the first questionnaire.

Your answers help us improve the course concept and to understand how students feel about analyzing their own genomic data.

Your participation in this questionnaire is voluntary. You may choose not to participate. If you choose to participate, you may stop at any time without any penalty. This will not affect your participation, grade, or any other aspect of your involvement in the course, or any other aspect of your education at HPI.

The procedure involves filling out a survey that will take approximately 20 minutes. Your survey data will be identified only by a random identifier; your name and other information that could identify you will not be on the questionnaires.

Please select your choice below:

- ○ I wish to continue with the questionnaire
- ○ I DO NOT wish to continue and want to exit

If you have any questions, concerns, or complaints at any time about this research please contact **aypgtteam@lists.myhpi.de**.

The questionnaire starts now, thank you for your participation.

General Information

On this page, you will find some information about the questionnaire in general.
Please read it carefully.

Regarding the question format:

- We kindly ask you to specify your answers if text fields are displayed.
- For most questions, you can select the options *I don't understand* and *I don't know*.
  - Select *I don't understand* if you do not understand the question or answer possibility.
  - Select *I don't know* if you understand the question and the answer possibilities but you cannot decide for an answer.

Throughout the questionnaire, technical terms appear that are defined as follows:

- **Genetic variations** or **variants**: A term used to describe the variation in the DNA sequence in each of our genomes. There are different ways that one person’s DNA sequence can differ from the reference DNA sequence. The most common type are so-called single nucleotide polymorphisms (SNPs).
- **Genotyping** is the process of determining which genetic variants an individual possesses. Genotyping can be performed through a variety of different methods, depending on the variants of interest and the resources available. For looking at many different variants at once, especially common variants, **genotyping arrays** are an efficient and accurate method. However, they require prior identification of the variants of interest.
- **Sequencing** is a method used to determine the exact sequence of a certain length of DNA. You can sequence a short piece, the whole genome, or parts of the genome, such as the exome, which are the regions of the genome that contain the instructions for RNA and proteins. Depending on the region, a given stretch of sequence may include some DNA that varies between individuals, in addition to regions that are constant. Thus, sequencing can be used to genotype someone for known variants, as well as identify variants that may be unique to that person.

Note: If not stated otherwise, we refer to array technology in questions concerning genotyping.

Before You Start

As a baseline for us to evaluate your further answers, please answer the question below.

1. Please state whether you understood the following concepts:

	Definitely yes	Probably yes	Neutral	Probably no	Definitely no	I prefer not to say
Variant	○	○	○	○	○	○
Genotyping	○	○	○	○	○	○
Array technology	○	○	○	○	○	○
Whole genome sequencing	○	○	○	○	○	○
The difference betreen array technology and whole genome sequencing	○	○	○	○	○	○

Course Motivation

We want to learn about your motivation to participate in this course.

2. Why did you enroll for the course?

	I strongly agree	I agree	Neutral	I disagree	I strongly disagree	I don't understand	I don't know	I prefer not to say
I want to receive my own genomic data	○	○	○	○	○	○	○	○
I want to learn about genome analysis	○	○	○	○	○	○	○	○
I want to analyze my own data	○	○	○	○	○	○	○	○
I need the credit points	○	○	○	○	○	○	○	○
The course sounded interesting	○	○	○	○	○	○	○	○

3. Are there other reasons why you enrolled for the course that are not covered above?

Course Expectations

We would like to know what you expect to learn in the AYPG course.

4. Please answer whether you expect to learn about the following topics.

	Definitely yes	Probably yes	Neutral	Probably no	Definitely no	I don’t understand	I don’t know	I prefer not to say
Genome-wide association studies	○	○	○	○	○	○	○	○
Ethical implications of genome analysis	○	○	○	○	○	○	○	○
Legal foundations of genome analysis in Germany	○	○	○	○	○	○	○	○
Comparison of legal foundations of genome analysis in Germany to other countries	○	○	○	○	○	○	○	○
Introduction to genotyping	○	○	○	○	○	○	○	○
Introduction to sequencing	○	○	○	○	○	○	○	○
Social implications of genome analysis	○	○	○	○	○	○	○	○
Pharmacogenomics	○	○	○	○	○	○	○	○
Ancestry analysis	○	○	○	○	○	○	○	○
Hands-on work with genetic data	○	○	○	○	○	○	○	○
Options for direct to consumer testing (in Germany and world-wide)	○	○	○	○	○	○	○	○
Analyzing wellness-traits	○	○	○	○	○	○	○	○
Tools to analyze and interpret (my own) genomic data	○	○	○	○	○	○	○	○

5. Are there other topics you expect to learn about that are not covered above?

6. **Are there topics in the context of genome analysis you do <u>not</u> expect to be covered but you want to learn about?**

Usage of Your Own Genomic Data (1 of 3)

We would like to know whether you intend to use your own genomic data, and if so, which analyses you plan to perform.

7. Did you get yourself already genotyped outside of this course?

- ○ Yes
- ○ No
- ○ I prefer not to say (because [ ])

8. Do you plan to get yourself genotyped?

- ○ Definitely yes
- ○ Probably yes
- ○ Probably no
- ○ Definitely no
- ○ It depends (on [ ])
- ○ I don’t understand
- ○ I don’t know
- ○ I prefer not to say (because [ ])

9. Would you discuss personal genotyping with someone or not prior to getting yourself genotyped?

[ ]

If yes, please tell us with whom (in terms of your personal or professional relationship rather than the person’s name). If no, please specify why.

10. At the present time, which of the following options would you prefer? Please check one.

- ○ **Option 1**: I would like to analyze my own genome
- ○ **Option 2**: I do **not** want to analyze my own genome and would rather analyze anonymous genomic data
- ○ I did not make any decision yet

11. In light of your choice to analyze your own genome or not, please respond to the following statements:

	I strongly agree	I agree	Neutral	I disagree	I strongly disagree	I don't understand	I don't know	I prefer not to say
I know which options are available to me	○	○	○	○	○	○	○	○
I know the benefits of each option	○	○	○	○	○	○	○	○
I know the risks of each option	○	○	○	○	○	○	○	○
I am clear about which risks and benefits matter most to me	○	○	○	○	○	○	○	○
I feel I have made an informed choice	○	○	○	○	○	○	○	○
I expect to stick with my decision	○	○	○	○	○	○	○	○
I am satisfied with my decision	○	○	○	○	○	○	○	○

Usage of Your Own Genomic Data (2 of 3)

We would like to know whether you intend to use your own genomic data, and if so, which analyses you plan to perform.

12. Which of the analyses below do you plan to conduct with your own data?

	Definitely yes	Probably yes	Neutral	Probably no	Definitely no	I don't understand	I don't know	I prefer not to say
I am not interested in my own data. I want to learn how to prepare and analyze genetic data for research in general	○	○	○	○	○	○	○	○
I plan to perform variant annotation of my own data, i.e. database queries to find out the effect or function of a genetic variant	○	○	○	○	○	○	○	○
I want to assess my personal disease risk using my own data	○	○	○	○	○	○	○	○
I want to calculate my genetic ancestry	○	○	○	○	○	○	○	○
I plan to calculate genetic scores, e.g. for wellness traits like eye color or male pattern baldness, using my own data	○	○	○	○	○	○	○	○
I plan to explore my pharmacogenomic markers, i.e. how my genes affect my response to drugs	○	○	○	○	○	○	○	○

13. What is your opinion regarding the following reasons <u>in favor of</u> genome analysis in the context of this course?

	I strongly agree	I agree	Neutral	I disagree	I strongly disagree	I don’t understand	I don’t know	I prefer not to say
To satisfy general curiosity	○	○	○	○	○	○	○	○
To inform family members about health risks	○	○	○	○	○	○	○	○
To understand what a patient may learn/experience	○	○	○	○	○	○	○	○
To help understand principles of human genetics	○	○	○	○	○	○	○	○

14. What is your opinion regarding the following reasons <u>against</u> genome analysis in the context of this course?

	I strongly agree	I agree	Neutral	I disagree	I strongly disagree	I don’t understand	I don’t know	I prefer not to say
Results are not accurate (the results are error-prone)	○	○	○	○	○	○	○	○
Results are not predictive (I cannot be sure a condition indicated by the results will break out)	○	○	○	○	○	○	○	○
I have concerns about privacy	○	○	○	○	○	○	○	○
Information will not be medically useful and will not change medical decisions	○	○	○	○	○	○	○	○
I get unwanted information	○	○	○	○	○	○	○	○

15. What is your opinion regarding genotyping as part of the course?

	I strongly agree	I agree	Neutral	I disagree	I strongly disagree	I don't understand	I don't know	I prefer not to say
I feel that I would be at a disadvantage to my classmates if I did not undergo the testing								
I see this as an opportunity to get a service that I would not ordinarily get								
I am concerned that my professors or course instructors would know who took up the offer of testing and who did not								
I am concerned that my classmates would know who took up the offer of testing and who did not								
I see this as an opportunity to get information that would help me improve my health and wellbeing								
I am concerned that I might get some results that would be disturbing								
I would only take up the offer of testing if I could get genetic counseling after I got my results back								
I would only take up the offer of testing if I could get genetic counseling before my sample is sent in								
I would be concerned that people would find out genetic or health information about me								

Usage of Your Own Genomic Data (3 of 3)

We would like to know whether you intend to use your own genomic data, and if so, which analyses you plan to perform.

16. If it was possible in Germany, would you get yourself genotyped with array technology if you had to pay for it yourself (around €60–100)

- Definitely yes
- Probably yes
- Probably no
- Definitely no
- It depends (on [   ])
- I don't understand
- I don't know
- I prefer not to say (because [   ])

17. In the context of this course, we are only allowed to show you a fraction of your data. After the course, you can ask for a password to access your complete genomic data set.

Are you planning to receive and analyze your complete data beyond the course?

- Iplan to further explore my genomic data beyond the course
- I want to receive my complete data, but I do not think I will look into it
- I do not want to receive my complete data
- I don't know
- I prefer not to say

18. Please explain your decision regarding the above question, on whether you would receive and analyze your complete data:

[   ]

19. I feel adequately trained to analyze and interpret my personal genomic data beyond the course.

- Definitely yes
- Probably yes
- Probably no
- Definitely no
- It depends (on)
- I don’t understand
- I don’t know
- I prefer not to say (because)

Data Usefulness

We would like to know how useful you think genotyping based on array technology is in the field, based on your current knowledge.

20. Please express your opinion on the following statements:

	I strongly agree	I agree	Neutral	I disagree	I strongly disagree	I don't understand	I don't know	I prefer not to say
I think the results from array genotyping are useful to physicians	○	○	○	○	○	○	○	○
I think the results from array genotyping are useful to patients themselves	○	○	○	○	○	○	○	○
I think the results from array genotyping lead to changes in patients' behavior	○	○	○	○	○	○	○	○
I think analyzing my own genomic data as part of the AYPG course would be useful	○	○	○	○	○	○	○	○
I think the results from array genotyping will lead to changes in my personal behavior	○	○	○	○	○	○	○	○
I think the results from array genotyping will lead to changes in my future health care decisions	○	○	○	○	○	○	○	○

Genome Analysis Regulations

This section assesses your opinion on regulations regarding genome analysis in Germany.

21. The German Genetic Diagnostics Act (Gendiagnostikgesetz, GenDG) states the following regulations:

- **Since 1 February 2010, anyone applying for human genetic analyses is obliged to comply with the new GenDG. The law regulates genetic testing for medical purposes, for determining parentage, for insurance purposes, and in the workplace.**
- **All genetic analyses must be accompanied by a detailed explanation of the nature, significance, and scope of the test, as well as a written patient consent. For prenatal and predictive analyses, supplementary genetic counseling must be recommended.**
- **Doctor's reservation (Arztvorbehalt) applies, i.e.:**
  - **All medical doctors are allowed to order a genetic test for disease diagnosis.**
  - **Only specialized medical doctors with human genetic training may order a genetic test to clarify disease risk.**

The text of the law can be found here.

In agreement with the GenDG, we will only work with a selected set of genetic markers during this course, e.g., genetic markers used for assessing "wellness traits" or genetic ancestry.

In light of this, please share your thoughts on the following:

	I strongly agree	I agree	Neutral	I disagree	I strongly disagree	I don't understand	I don't know	I prefer not to say
I think the GenDG is appropriate	○	○	○	○	○	○	○	○
I think genetic testing should be allowed independently of disease diagnosis	○	○	○	○	○	○	○	○
I think genetic testing should be allowed without genetic counseling	○	○	○	○	○	○	○	○
In the context of clinical diagnostics, I would make use of genetic counseling	○	○	○	○	○	○	○	○
I would like to analyze my complete data	○	○	○	○	○	○	○	○
I would like to analyze only parts of my data	○	○	○	○	○	○	○	○
I would only like to receive my complete data, but I do not want to analyze it	○	○	○	○	○	○	○	○

22. If you would like to analyze only parts of your data, please specify which parts:

23. Do you have other thoughts on the GenDG you want to share with us?

Whole Genome Sequencing

Thus far, we have offered genotyping using array technology in the AYPG course, which can only detect specific variants. However, in this section we would like to hear your opinion on **whole genome sequencing**.

24. What is your opinion regarding whole genome sequencing (WGS)?

	I strongly agree	I agree	Neutral	I disagree	I strongly disagree	I don't understand	I don't know	I prefer not to say
I think WGS results are more accurate than results from array data	○	○	○	○	○	○	○	○
I think WGS results are more predictive than results from array data	○	○	○	○	○	○	○	○
I would prefer WGS technology over array technology for genotyping my own data	○	○	○	○	○	○	○	○
I think WGS results are connected with more risks than results from array data	○	○	○	○	○	○	○	○
I am more concerned about privacy issues when using WGS technology, compared to using array technology	○	○	○	○	○	○	○	○
I think the results from WGS are more useful to a physician than results from array data	○	○	○	○	○	○	○	○
I think the results from WGS are more useful to patients themselves than results from array data	○	○	○	○	○	○	○	○
I think the results from WGS would more likely lead to changes in patients' behavior than results from array data	○	○	○	○	○	○	○	○
I think the results from WGS would more likely lead to changes in my personal behavior than results from array data	○	○	○	○	○	○	○	○
I think the results from WGS would more likely lead to changes in my future health care decisions than results from array data	○	○	○	○	○	○	○	○

25. Did you get your genome sequenced with WGS outside of this course?

- Yes
- No
- I prefer not to say (because ______)

26. If it was possible in Germany, would you get yourself genotyped with WGS at no charge?

- Definitely yes
- Probably yes
- Probably no
- Definitely no
- It depends (on ______)
- I don’t understand
- I don’t know
- I prefer not to say (because ______)

27. If it was possible in Germany, would you get yourself genotyped with WGS if you had to pay for it yourself (around €600–1000)?

- Definitely yes
- Probably yes
- Probably no
- Definitely no
- It depends (on ______)
- I don’t understand
- I don’t know
- I prefer not to say (because ______)

Page 12
Outro complete

End of Course Questionnaire

A big **thank you** for helping us to improve the AYPG lecture and to learn about your attitudes towards genome analysis.

If you have any further comments, please leave them here:

Page 13
Outro canceled

Feedback

It is totally fine that you do not want to continue with the questionnaire. It would be great if you could tell us why and help us to improve.

Please explain why you did not participate. If you have any further comments, please leave them here as well:

Thank you for completing this questionnaire!

Your answers were transmitted, you may close the browser window or tab now.

HPI 2020 Q2

Questionnaire 2: Mid-Course

Analyse Your Personal Genome (AYPG)

Dear AYPG student,

The purpose of this questionnaire is to learn about your expectations of the course and your opinion regarding analyzing your own genome and genome analysis in general. In total, we will ask you to answer three consecutive questionnaires at different time points in the course.

This is the second questionnaire.

Your answers help us improve the course concept and to understand how students feel about analyzing their own genomic data.

Your participation in this questionnaire is voluntary. You may choose not to participate. If you choose to participate, you may stop at any time without any penalty. This will not affect your participation, grade, or any other aspect of your involvement in the course, or any other aspect of your education at HPI.

The procedure involves filling out a survey that will take approximately 20 minutes. Your survey data will be identified only by a random identifier; your name and other information that could identify you will not be on the questionnaires.

Please select your choice below:

- ○ I wish to continue with the questionnaire
- ○ I DO NOT wish to continue and want to exit

If you have any questions, concerns, or complaints at any time about this research please contact **aypgtteam@lists.myhpi.de**.

The questionnaire starts now, thank you for your participation.

General Information

On this page, you will find some information about the questionnaire in general.
Please read it carefully.

Regarding the question format:

- We kindly ask you to specify your answers if text fields are displayed.
- For most questions, you can select the options *I don't understand* and *I don't know*.
  - Select *I don't understand* if you do not understand the question or answer possibility.
  - Select *I don't know* if you understand the question and the answer possibilities but you cannot decide for an answer.

Throughout the questionnaire, technical terms appear that are defined as follows:

- **Genetic variations** or **variants**: A term used to describe the variation in the DNA sequence in each of our genomes. There are different ways that one person's DNA sequence can differ from the reference DNA sequence. The most common type are so-called single nucleotide polymorphisms (SNPs).
- **Genotyping** is the process of determining which genetic variants an individual possesses. Genotyping can be performed through a variety of different methods, depending on the variants of interest and the resources available. For looking at many different variants at once, especially common variants, **genotyping arrays** are an efficient and accurate method. However, they require prior identification of the variants of interest.
- **Sequencing** is a method used to determine the exact sequence of a certain length of DNA. You can sequence a short piece, the whole genome, or parts of the genome, such as the exome, which are the regions of the genome that contain the instructions for RNA and proteins. Depending on the region, a given stretch of sequence may include some DNA that varies between individuals, in addition to regions that are constant. Thus, sequencing can be used to genotype someone for known variants, as well as identify variants that may be unique to that person.

Note: If not stated otherwise, we refer to array technology in questions concerning genotyping.

Before You Start

As a baseline for us to evaluate your further answers, please answer the question below.

1. Please state whether you understood the following concepts:

	Definitely yes	Probably yes	Neutral	Probably no	Definitely no	I prefer not to say
Variant	○	○	○	○	○	○
Genotyping	○	○	○	○	○	○
Array technology	○	○	○	○	○	○
Whole genome sequencing	○	○	○	○	○	○
The difference betreen array technology and whole genome sequencing	○	○	○	○	○	○

Usage of Your Own Genomic Data (1 of 3)

We would like to know whether you intend to use your own genomic data, and if so, which analyses you plan to perform.

2. Did you get yourself genotyped as part of this course?

- ○ Yes
- ○ No
- ○ I prefer not to say (because ______)

3. Did you discuss whether or not to get yourself genotyped as part of this course with any of the following?

- ☐ Genetic counselor
- ☐ Physician or other health professional
- ☐ Parents
- ☐ Sibling(s)
- ☐ Other family members
- ☐ Friend(s)
- ☐ Spouse/significant other
- ☐ Course instructor(s)
- ☐ Other: ______
- ☐ I prefer not to say (because ______)

4. Please reflect on the decision that you made about receiving or not receiving your genomic data as part of the course.

28. Would you go for the same choice again?

- ○ Definitely yes
- ○ Probably yes
- ○ Probably no
- ○ Definitely no
- ○ It depends (on ______)
- ○ I don't understand
- ○ I don't know
- ○ I prefer not to say (because ______)

5. Regarding your decision whether or not to get yourself genotyped, please indicate to what extend each of the statements below is true to you <u>at the present time</u>:

	I strongly agree	I agree	Neutral	I disagree	I strongly disagree	I don't understand	I don't know	I prefer not to say
I feel adequately informed about the issues important to my decision	○	○	○	○	○	○	○	○
The decision I made was the best decision possible for me personally	○	○	○	○	○	○	○	○
I am satisfied that this was my decision to make	○	○	○	○	○	○	○	○
I am satisfied with my decision	○	○	○	○	○	○	○	○
I am comfortable with my decision	○	○	○	○	○	○	○	○

Usage of Your Own Genomic Data (2 of 3)

We would like to know whether you intend to use your own genomic data, and if so, which analyses you plan to perform.

6. Which of the analyses below do you plan to conduct with your own data?

	Definitely yes	Probably yes	Neutral	Probably no	Definitely no	I don't understand	I don't know	I prefer not to say
I am not interested in my own data. I want to learn how to prepare and analyze genetic data for research in general								
I plan to perform variant annotation of my own data, i.e. database queries to find out the effect or function of a genetic variant								
I want to assess my personal disease risk using my own data								
I want to calculate my genetic ancestry								
I plan to calculate genetic scores, e.g. for wellness traits like eye color or male pattern baldness, using my own data								
I plan to explore my pharmacogenomic markers, i.e. how my genes affect my response to drugs								

7. What is your opinion regarding the following reasons in favor of genome analysis in the context of this course?

	I strongly agree	I agree	Neutral	I disagree	I strongly disagree	I don't understand	I don't know	I prefer not to say
To satisfy general curiosity	○	○	○	○	○	○	○	○
To inform family members about health risks	○	○	○	○	○	○	○	○
To understand what a patient may learn/experience	○	○	○	○	○	○	○	○
To help understand principles of human genetics	○	○	○	○	○	○	○	○

8. What is your opinion regarding the following reasons against genome analysis in the context of this course?

	I strongly agree	I agree	Neutral	I disagree	I strongly disagree	I don't understand	I don't know	I prefer not to say
Results are not accurate (the results are error-prone)	○	○	○	○	○	○	○	○
Results are not predictive (I cannot be sure a condition indicated by the results will break out)	○	○	○	○	○	○	○	○
I have concerns about privacy	○	○	○	○	○	○	○	○
Information will not be medically useful and will not change medical decisions	○	○	○	○	○	○	○	○
I get unwanted information	○	○	○	○	○	○	○	○

9. What is your opinion regarding genotyping as part of the course?

	I strongly agree	I agree	Neutral	I disagree	I strongly disagree	I don't understand	I don't know	I prefer not to say
I feel that I would be at a disadvantage to my classmates if I did not undergo the testing	○	○	○	○	○	○	○	○
I see this as an opportunity to get a service that I would not ordinarily get	○	○	○	○	○	○	○	○
I am concerned that my professors or course instructors would know who took up the offer of testing and who did not	○	○	○	○	○	○	○	○
I am concerned that my classmates would know who took up the offer of testing and who did not	○	○	○	○	○	○	○	○
I see this as an opportunity to get information that would help me improve my health and wellbeing	○	○	○	○	○	○	○	○
I am concerned that I might get some results that would be disturbing	○	○	○	○	○	○	○	○
I would only take up the offer of testing if I could get genetic counseling after I got my results back	○	○	○	○	○	○	○	○
I would only take up the offer of testing if I could get genetic counseling before my sample is sent in	○	○	○	○	○	○	○	○
I would be concerned that people would find out genetic or health information about me	○	○	○	○	○	○	○	○

Usage of Your Own Genomic Data (3 of 3)

We would like to know whether you intend to use your own genomic data, and if so, which analyses you plan to perform.

29. If it was possible in Germany, would you get yourself genotyped with array technology if you had to pay for it yourself (around €60–100)?

- Definitely yes
- Probably yes
- Probably no
- Definitely no
- It depends (on ______)
- I don’t understand
- I don’t know
- I prefer not to say (because ______)

10. In the context of this course, we are only allowed to show you a fraction of your data. After the course, you can ask for a password to access your complete genomic data set.

Are you planning to receive and analyze your complete data beyond the course?

- Iplan to further explore my genomic data beyond the course
- I want to receive my complete data, but I do not think I will look into it
- I do not want to receive my complete data
- I don’t know
- I prefer not to say

11. Please explain your decision regarding the above question, on whether you would receive and analyze your complete data:

24. I feel adequately trained to analyze and interpret my personal genomic data beyond the course.

- Definitely yes
- Probably yes
- Probably no
- Definitely no
- It depends (on ______)
- I don’t understand
- I don’t know
- I prefer not to say (because ______)

Data Usefulness

We would like to know how useful you think genotyping based on array technology is in the field, based on your current knowledge.

14. Please express your opinion on the following statements:

	I strongly agree	I agree	Neutral	I disagree	I strongly disagree	I don't understand	I don't know	I prefer not to say
I think the results from array genotyping are useful to physicians								
I think the results from array genotyping are useful to patients themselves								
I think the results from array genotyping lead to changes in patients' behavior								
I think analyzing my own genomic data as part of the AYPG course would be useful								
I think the results from array genotyping will lead to changes in my personal behavior								
I think the results from array genotyping will lead to changes in my future health care decisions								

Genome Analysis Regulations

This section assesses your opinion on regulations regarding genome analysis in Germany.

15. The German Genetic Diagnostics Act (Gendiagnostikgesetz, GenDG) states the following regulations:

- **Since 1 February 2010, anyone applying for human genetic analyses is obliged to comply with the new GenDG. The law regulates genetic testing for medical purposes, for determining parentage, for insurance purposes, and in the workplace.**
- **All genetic analyses must be accompanied by a detailed explanation of the nature, significance, and scope of the test, as well as a written patient consent. For prenatal and predictive analyses, supplementary genetic counseling must be recommended.**
- **Doctor's reservation (Arztvorbehalt) applies, i.e.:**
  - **All medical doctors are allowed to order a genetic test for disease diagnosis.**
  - **Only specialized medical doctors with human genetic training may order a genetic test to clarify disease risk.**

The text of the law can be found here.

In agreement with the GenDG, we will only work with a selected set of genetic markers during this course, e.g., genetic markers used for assessing "wellness traits" or genetic ancestry.

In light of this, please share your thoughts on the following:

	I strongly agree	I agree	Neutral	I disagree	I strongly disagree	I don't understand	I don't know	I prefer not to say
I think the GenDG is appropriate								
I think genetic testing should be allowed independently of disease diagnosis								
I think genetic testing should be allowed without genetic counseling								
In the context of clinical diagnostics, I would make use of genetic counseling								
I would like to analyze my complete data								
I would like to analyze only parts of my data								
I would only like to receive my complete data, but I do not want to analyze it								

16. If you would like to analyze only parts of your data, please specify which parts:

17. Do you have other thoughts on the GenDG you want to share with us?

Whole Genome Sequencing

Thus far, we have offered genotyping using array technology in the AYPG course, which can only detect specific variants. However, in this section we would like to hear your opinion on **whole genome sequencing**.

18. What is your opinion regarding whole genome sequencing (WGS)?

	I strongly agree	I agree	Neutral	I disagree	I strongly disagree	I don't understand	I don't know	I prefer not to say
I think WGS results are more accurate than results from array data	○	○	○	○	○	○	○	○
I think WGS results are more predictive than results from array data	○	○	○	○	○	○	○	○
I would prefer WGS technology over array technology for genotyping my own data	○	○	○	○	○	○	○	○
I think WGS results are connected with more risks than results from array data	○	○	○	○	○	○	○	○
I am more concerned about privacy issues when using WGS technology, compared to using array technology	○	○	○	○	○	○	○	○
I think the results from WGS are more useful to a physician than results from array data	○	○	○	○	○	○	○	○
I think the results from WGS are more useful to patients themselves than results from array data	○	○	○	○	○	○	○	○
I think the results from WGS would more likely lead to changes in patients' behavior than results from array data	○	○	○	○	○	○	○	○
I think the results from WGS would more likely lead to changes in my personal behavior than results from array data	○	○	○	○	○	○	○	○
I think the results from WGS would more likely lead to changes in my future health care decisions than results from array data	○	○	○	○	○	○	○	○

19. Did you get your genome sequenced with WGS outside of this course?

- Yes
- No
- I prefer not to say (because ______)

30. If it was possible in Germany, would you get yourself genotyped with WGS at no charge?

- Definitely yes
- Probably yes
- Probably no
- Definitely no
- It depends (on ______)
- I don’t understand
- I don’t know
- I prefer not to say (because ______)

31. If it was possible in Germany, would you get yourself genotyped with WGS if you had to pay for it yourself (around €600–1000)?

- Definitely yes
- Probably yes
- Probably no
- Definitely no
- It depends (on ______)
- I don’t understand
- I don’t know
- I prefer not to say (because ______)

Intermediate Course Retrospective

In order to improve the Analyze Your Personal Genome course, we are extremely interested how you liked the course and the covered topics so far.

22. What worked well? Please tell us why.

23. What did not work well? Please tell us why.

Page 11
Outro complete

End of Course Questionnaire

A big **thank you** for helping us to improve the AYPG lecture and to learn about your attitudes towards genome analysis.

If you have any further comments, please leave them here:

Page 12
Outro canceled

Feedback

It is totally fine that you do not want to continue with the questionnaire. It would be great if you could tell us why and help us to improve.

Please explain why you did not participate. If you have any further comments, please leave them here as well:

Thank you for completing this questionnaire!

Your answers were transmitted, you may close the browser window or tab now.

HPI 2020 Q3

Questionnaire 3: End of Course

Analyse Your Personal Genome (AYPG)

Dear AYPG student,

The purpose of this questionnaire is to learn about your expectations of the course and your opinion regarding analyzing your own genome and genome analysis in general. In total, we will ask you to answer three consecutive questionnaires at different time points in the course.

This is the third questionnaire.

Your answers help us improve the course concept and to understand how students feel about analyzing their own genomic data.

Your participation in this questionnaire is voluntary. You may choose not to participate. If you choose to participate, you may stop at any time without any penalty. This will not affect your participation, grade, or any other aspect of your involvement in the course, or any other aspect of your education at HPI.

The procedure involves filling out a survey that will take approximately 20 minutes. Your survey data will be identified only by a random identifier; your name and other information that could identify you will not be on the questionnaires.

Please select your choice below:

- ○ I wish to continue with the questionnaire
- ○ I DO NOT wish to continue and want to exit

If you have any questions, concerns, or complaints at any time about this research please contact **aypgtteam@lists.myhpi.de**.

The questionnaire starts now, thank you for your participation.

General Information

On this page, you will find some information about the questionnaire in general.
Please read it carefully.

Regarding the question format:

- We kindly ask you to specify your answers if text fields are displayed.
- For most questions, you can select the options *I don't understand* and *I don't know*.
  - Select *I don't understand* if you do not understand the question or answer possibility.
  - Select *I don't know* if you understand the question and the answer possibilities but you cannot decide for an answer.

Throughout the questionnaire, technical terms appear that are defined as follows:

- **Genetic variations** or **variants**: A term used to describe the variation in the DNA sequence in each of our genomes. There are different ways that one person's DNA sequence can differ from the reference DNA sequence. The most common type are so-called single nucleotide polymorphisms (SNPs).
- **Genotyping** is the process of determining which genetic variants an individual possesses. Genotyping can be performed through a variety of different methods, depending on the variants of interest and the resources available. For looking at many different variants at once, especially common variants, **genotyping arrays** are an efficient and accurate method. However, they require prior identification of the variants of interest.
- **Sequencing** is a method used to determine the exact sequence of a certain length of DNA. You can sequence a short piece, the whole genome, or parts of the genome, such as the exome, which are the regions of the genome that contain the instructions for RNA and proteins. Depending on the region, a given stretch of sequence may include some DNA that varies between individuals, in addition to regions that are constant. Thus, sequencing can be used to genotype someone for known variants, as well as identify variants that may be unique to that person.

Note: If not stated otherwise, we refer to array technology in questions concerning genotyping.

Before You Start

As a baseline for us to evaluate your further answers, please answer the question below.

1. Please state whether you understood the following concepts:

	Definitely yes	Probably yes	Neutral	Probably no	Definitely no	I prefer not to say
Variant	○	○	○	○	○	○
Genotyping	○	○	○	○	○	○
Array technology	○	○	○	○	○	○
Whole genome sequencing	○	○	○	○	○	○
The difference betreen array technology and whole genome sequencing	○	○	○	○	○	○

Usage of Your Own Genomic Data (1 of 3)

We would like to know whether you intend to use your own genomic data, and if so, which analyses you plan to perform.

2. Did you get yourself genotyped as part of this course?

- ○ Yes
- ○ No
- ○ I prefer not to say (because ______)

3. Did you discuss whether or not to get yourself genotyped as part of this course with any of the following?

- ☐ Genetic counselor
- ☐ Physician or other health professional
- ☐ Parents
- ☐ Sibling(s)
- ☐ Other family members
- ☐ Friend(s)
- ☐ Spouse/significant other
- ☐ Course instructor(s)
- ☐ Other: ______
- ☐ I prefer not to say (because ______)

4. Please reflect on the decision that you made about receiving or not receiving your genomic data as part of the course.

Would you go for the same choice again?

- ○ Definitely yes
- ○ Probably yes
- ○ Probably no
- ○ Definitely no
- ○ It depends (on [ ])
- ○ I don't understand
- ○ I don't know
- ○ I prefer not to say (because [ ])

5. Regarding your decision whether or not to get yourself genotyped, please indicate to what extend each of the statements below is true to you <u>at the present time</u>:

	I strongly agree	I agree	Neutral	I disagree	I strongly disagree	I don't understand	I don't know	I prefer not to say
I feel adequately informed about the issues important to my decision	○	○	○	○	○	○	○	○
The decision I made was the best decision possible for me personally	○	○	○	○	○	○	○	○
I am satisfied that this was my decision to make	○	○	○	○	○	○	○	○
I am satisfied with my decision	○	○	○	○	○	○	○	○
I am comfortable with my decision	○	○	○	○	○	○	○	○

Usage of Your Own Genomic Data (2 of 3)

We would like to know whether you intend to use your own genomic data, and if so, which analyses you plan to perform.

6. Which of the analyses below did you conduct with your own data?

- ☐ Perform variant annotation of my own data, i.e. database queries to find out the effect or function of a genetic variant
- ☐ Assessing my personal disease risk using my own data
- ☐ Calculate my genetic ancestry
- ☐ Calculate genetic scores, e.g. for wellness traits like eye color or male pattern baldness, using my own data
- ☐ Explore my pharmacogenomic markers, i.e. how my genes affect my response to drugs
- ☐ Other: ______
- ☐ I prefer not to say (because ______)

7. What is your opinion regarding the following reasons in favor of genome analysis in the context of this course?

	I strongly agree	I agree	Neutral	I disagree	I strongly disagree	I don't understand	I don't know	I prefer not to say
To satisfy general curiosity	○	○	○	○	○	○	○	○
To inform family members about health risks	○	○	○	○	○	○	○	○
To understand what a patient may learn/experience	○	○	○	○	○	○	○	○
To help understand principles of human genetics	○	○	○	○	○	○	○	○

8. What is your opinion regarding the following reasons against genome analysis in the context of this course?

	I strongly agree	I agree	Neutral	I disagree	I strongly disagree	I don't understand	I don't know	I prefer not to say
Results are not accurate (the results are error-prone)	○	○	○	○	○	○	○	○
Results are not predictive (I cannot be sure a condition indicated by the results will break out)	○	○	○	○	○	○	○	○
I have concerns about privacy	○	○	○	○	○	○	○	○
Information will not be medically useful and will not change medical decisions	○	○	○	○	○	○	○	○
I get unwanted information	○	○	○	○	○	○	○	○

9. What is your opinion regarding genotyping as part of the course?

	I strongly agree	I agree	Neutral	I disagree	I strongly disagree	I don't understand	I don't know	I prefer not to say
I feel that I would be at a disadvantage to my classmates if I did not undergo the testing	○	○	○	○	○	○	○	○
I see this as an opportunity to get a service that I would not ordinarily get	○	○	○	○	○	○	○	○
I am concerned that my professors or course instructors would know who took up the offer of testing and who did not	○	○	○	○	○	○	○	○
I am concerned that my classmates would know who took up the offer of testing and who did not	○	○	○	○	○	○	○	○
I see this as an opportunity to get information that would help me improve my health and wellbeing	○	○	○	○	○	○	○	○
I am concerned that I might get some results that would be disturbing	○	○	○	○	○	○	○	○
I would only take up the offer of testing if I could get genetic counseling after I got my results back	○	○	○	○	○	○	○	○
I would only take up the offer of testing if I could get genetic counseling before my sample is sent in	○	○	○	○	○	○	○	○
I would be concerned that people would find out genetic or health information about me	○	○	○	○	○	○	○	○

Usage of Your Own Genomic Data (3 of 3)

We would like to know whether you intend to use your own genomic data, and if so, which analyses you plan to perform.

32. If it was possible in Germany, would you get yourself genotyped with array technology if you had to pay for it yourself (around €60–100)

- ○ Definitely yes
- ○ Probably yes
- ○ Probably no
- ○ Definitely no
- ○ It depends (on ______)
- ○ I don’t understand
- ○ I don’t know
- ○ I prefer not to say (because ______)

10. In the context of this course, we are only allowed to show you a fraction of your data. After the course, you can ask for a password to access your complete genomic data set.

Are you planning to receive and analyze your complete data beyond the course?

- ○ Iplan to further explore my genomic data beyond the course
- ○ I want to receive my complete data, but I do not think I will look into it
- ○ I do not want to receive my complete data
- ○ I don’t know
- ○ I prefer not to say

11. Please explain your decision regarding the above question, on whether you would receive and analyze your complete data:

33. I feel adequately trained to analyze and interpret my personal genomic data beyond the course.

- ◯ Definitely yes
- ◯ Probably yes
- ◯ Probably no
- ◯ Definitely no
- ◯ It depends (on ______)
- ◯ I don’t understand
- ◯ I don’t know
- ◯ I prefer not to say (because ______)

Data Usefulness

We would like to know how useful you think genotyping based on array technology is in the field, based on your current knowledge.

14. Please express your opinion on the statements:

	I strongly agree	I agree	Neutral	I disagree	I strongly disagree	I don't understand	I don't know	I prefer not to say
I think the results from array genotyping are useful to physicians	○	○	○	○	○	○	○	○
I think the results from array genotyping are useful to patients themselves	○	○	○	○	○	○	○	○
I think the results from array genotyping lead to changes in patients' behavior	○	○	○	○	○	○	○	○
I think analyzing my own genomic data as part of the AYPG course was useful	○	○	○	○	○	○	○	○
I think the results from array genotyping will lead to changes in my personal behavior	○	○	○	○	○	○	○	○
I think the results from array genotyping will lead to changes in my future health care decisions	○	○	○	○	○	○	○	○

Genome Analysis Regulations

This section assesses your opinion on regulations regarding genome analysis in Germany.

15. The German Genetic Diagnostics Act (Gendiagnostikgesetz, GenDG) states the following regulations:

- **Since 1 February 2010, anyone applying for human genetic analyses is obliged to comply with the new GenDG. The law regulates genetic testing for medical purposes, for determining parentage, for insurance purposes, and in the workplace.**
- **All genetic analyses must be accompanied by a detailed explanation of the nature, significance, and scope of the test, as well as a written patient consent. For prenatal and predictive analyses, supplementary genetic counseling must be recommended.**
- **Doctor's reservation (Arztvorbehalt) applies, i.e.:**
  - **All medical doctors are allowed to order a genetic test for disease diagnosis.**
  - **Only specialized medical doctors with human genetic training may order a genetic test to clarify disease risk.**

The text of the law can be found here.

In agreement with the GenDG, we will only work with a selected set of genetic markers during this course, e.g., genetic markers used for assessing "wellness traits" or genetic ancestry.

In light of this, please share your thoughts on the following:

	I strongly agree	I agree	Neutral	I disagree	I strongly disagree	I don't understand	I don't know	I prefer not to say
I think the GenDG is appropriate								
I think genetic testing should be allowed independently of disease diagnosis								
I think genetic testing should be allowed without genetic counseling								
In the context of clinical diagnostics, I would make use of genetic counseling								
I would like to analyze my complete data								
I would like to analyze only parts of my data								
I would only like to receive my complete data, but I do not want to analyze it								

16. If you would like to analyze only parts of your data, please specify which parts:

17. Do you have other thoughts on the GenDG you want to share with us?

Whole Genome Sequencing

Thus far, we have offered genotyping using array technology in the AYPG course, which can only detect specific variants. However, in this section we would like to hear your opinion on **whole genome sequencing**.

18. What is your opinion regarding whole genome sequencing (WGS)?

	I strongly agree	I agree	Neutral	I disagree	I strongly disagree	I don't understand	I don't know	I prefer not to say
I think WGS results are more accurate than results from array data	○	○	○	○	○	○	○	○
I think WGS results are more predictive than results from array data	○	○	○	○	○	○	○	○
I would prefer WGS technology over array technology for genotyping my own data	○	○	○	○	○	○	○	○
I think WGS results are connected with more risks than results from array data	○	○	○	○	○	○	○	○
I am more concerned about privacy issues when using WGS technology, compared to using array technology	○	○	○	○	○	○	○	○
I think the results from WGS are more useful to a physician than results from array data	○	○	○	○	○	○	○	○
I think the results from WGS are more useful to patients themselves than results from array data	○	○	○	○	○	○	○	○
I think the results from WGS would more likely lead to changes in patients' behavior than results from array data	○	○	○	○	○	○	○	○
I think the results from WGS would more likely lead to changes in my personal behavior than results from array data	○	○	○	○	○	○	○	○
I think the results from WGS would more likely lead to changes in my future health care decisions than results from array data	○	○	○	○	○	○	○	○

19. Did you get your genome sequenced with WGS outside of this course?

- ○ Yes
- ○ No
- ○ I prefer not to say (because [ ])

34. If it was possible in Germany, would you get yourself genotyped with WGS at no charge?

- ○ Definitely yes
- ○ Probably yes
- ○ Probably no
- ○ Definitely no
- ○ It depends (on [ ])
- ○ I don't understand
- ○ I don't know
- ○ I prefer not to say (because [ ])

35. If it was possible in Germany, would you get yourself genotyped with WGS if you had to pay for it yourself (around €600–1000)?

- ○ Definitely yes
- ○ Probably yes
- ○ Probably no
- ○ Definitely no
- ○ It depends (on [ ])
- ○ I don't understand
- ○ I don't know
- ○ I prefer not to say (because [ ])

Course Retrospective

In order to improve the Analyze Your Personal Genome course, we are extremely interested how you liked the course and the covered topics.

22. What worked well? Please tell us why.

23. What did not work well? Please tell us why.

Page 11
Outro complete

End of Course Questionnaire

A big **thank you** for helping us to improve the AYPG lecture and to learn about your attitudes towards genome analysis.

If you have any further comments, please leave them here:

Page 12
Outro canceled

Feedback

It is totally fine that you do not want to continue with the questionnaire. It would be great if you could tell us why and help us to improve.

Please explain why you did not participate. If you have any further comments, please leave them here as well:

Thank you for completing this questionnaire!

Your answers were transmitted, you may close the browser window or tab now.

HPI 2020 Q4

Analyze Your Personal Genome

Retrospective Questionnaire

The purpose of this questionnaire is to compare students' attitudes regarding personal genotyping over the course. The survey is conducted in the courses *Genomische Medizin* at the Technical University of Munich and *Analyze Your Personal Genome* at Hasso Plattner Institute in Potsdam. Filling out this questionnaire will take less than 5 minutes.

Your participation in this questionnaire is anonymous to the teaching staff. Your name or other information that could identify you will not be on the questionnaires. Your responses will only be identified by a pseudonym so that we can follow the development of answers throughout questionnaires. Only the course honest broker knows the mapping of pseudonyms and names and has no access to the pseudonymized results. We will anonymize the data by deleting the mapping and additionally assigning new, random pseudonyms.

Your participation in this questionnaire is voluntary. You may choose not to participate. If you choose to participate, you may stop at any time without any penalty. This will not affect your participation, grade, or any other aspect of your involvement in the course, or any other aspect of your education at HPI.

We plan to publish the results of this survey in a scientific journal. We will only use anonymous data for the publication and we will not cite free text answers.

By continuing with the questionnaire, you confirm that you have read and understood the information above and agree to the conditions for participation.

- ○ I want to participate in the study
- ○ I do <u>not</u> want to participate in the study and want to exit

Page 02

1. Did you study medicine?

- Yes
- No

Page 03

2. What motivated you to participate in the course?

- General interest in genomics/genomic analyses
- To receive/analyze my own genomic data
- To gain knowledge about genomics/genomic analyses to apply it in my professional career
- To receive the credit points
- To learn about tools for variant interpretation and analysis
- Interest in ancestry analysis
- Interest in pharmacogenomics (e.g., the impact of genomic variants on medication effects)
- Interest in research topics like genome-wide association studies
- Interest in commercial genomic testing ("direct-to-consumer testing")
- To better understand the situation of patients when undergoing genomic testing
- Interest in ethical issues in the context of genomic analyses
- Interest in legal foundations of genomic analyses

Page 04

3. Did you participate in personal genotyping as part of the course?

- Yes
- No

Page 05

4. Has this course changed your attitude towards personal genotype analysis?

- Yes
- No

- I don’t know

Page 06

5. How did the course change your attitude?

- Through the course I have gained a more positive opinion on genetic analysis
- Through the course I have become more critical about genetic analysis

Page 07

6. Would you have participated in personal genotyping as part of the course if it had been offered via a non-european private company, such as 23andMe?

- Yes
- No

Page 08

7. Did you collect the password for your complete genotype data?

- Yes
- No

Page 09

8. Do you still plan to collect the password for your complete genomic data?

- Yes
- No

Page 10

9. Did you conduct analyses with your own data beyond the course?

- Analyzing genetic ancestry
- Analyzing pharmacogenomic markers (medication effects)
- Analyzing wellness traits (not disease-relevant, e.g., eye color or blood type)
- Carrier detection of risk variants for monogenetic diseases
- Analyzing risk of (common) polygenic diseases
- No, I just wanted to receive my data

Page 11

10. Do you plan to conduct further analyses with your own genomic data?

- Yes
- No

Page 12

11. Would you recommend personal genotyping to relatives or friends who have not taken the course?

- Yes
- No

Page 13

12. Do you think that personal genotyping in the course context is useful for the learning experience?

- Yes
- No

Page 14

13. How important do you consider the treatment of ethical aspects in the AYPG course?

- Absolutely necessary
- Rather important
- Neutral
- Rather unimportant
- Completely unimportant

Page 15

14. Would you recommend to offer personal genotyping in future courses again?

- Yes
- No

Page 16

15. Should personal genotyping be allowed without genetic counseling by a medical professional?

- Yes
- No

Page 17

16. Do you feel adequately trained to analyze and interpret genomic data beyond the course?

- Yes
- No

Last Page

Thank you for your time!

Your answers were transmitted, you may close the browser window or tab now.

TUM Q4‘

Seite 01

Genomische Medizin

Retrospektiver Fragebogen

Das Ziel dieser Umfrage ist es, rückblickend die Einstellung von Teilnehmern des Kurses *Genomische Medizin* an der Technischen Universität München gegenüber Genomanalysen zu erfassen. Ähnliche Umfragen werden im derzeit stattfindenden Kurs und im Kurs *Analyze Your Personal Genome* am Hasso-Plattner-Institut in Potsdam durchgeführt. Die Beantwortung dieses Fragebogens dauert etwa 5 Minuten.

Die Teilnahme an diesem Fragebogen ist anonym. Wir speichern keine persönlichen Daten.

Die Teilnahme an diesem Fragebogen ist freiwillig. Sie können sich dafür entscheiden, nicht teilzunehmen.

Wir planen, die Ergebnisse dieser Umfrage anonymisiert zu publizieren. Wir werden für die wissenschaftliche Veröffentlichung nur aggregierte Daten verwenden, die keine Rückschlüsse auf die Teilnehmer erlauben.

Wenn Sie mit dem Fragebogen fortfahren, stimmen Sie damit den oben genannten Bedingungen zu.

- ○ Ich möchte an der Umfrage teilnehmen
- ○ Ich möchte nicht an der Umfrage teilnehmen und möchte die Umfrage beenden

---

Seite 02

1. Wann haben Sie an dem Kurs teilgenommen?

[Bitte auswählen]

Optionen: SoSe 2017, WiSe 2017/18, SoSe 2018, WiSe 2018/19, WiSe 2019/20

Seite 03

2. Was haben Sie studiert oder welche Ausbildung hatten Sie, als Sie den Kurs belegt haben?

[Bitte auswählen]

Optionen: Medicine, Genetic and Genomic Counseling, Other educational background

Seite 04

3. Was hat Sie motiviert, den Kurs zu belegen?

- ☐ Allgemeines Interesse am Thema Genetik/Genomanalysen
- ☐ Um meine eigenen genetischen Daten zu erhalten/analysieren
- ☐ Um Wissen über Genetik/Genomanalysen zu erlangen und dieses in meinem Beruf einzusetzen
- ☐ Um Werkzeuge kennenzulernen, mit denen ich genetische Daten interpretieren und analysieren kann
- ☐ Interesse an genetischen Abstammungsanalysen
- ☐ Interesse an Pharmakogenomik (z.B. der Auswirkung von genetischen Varianten auf die Medikamentenwirkung)
- ☐ Interesse an Forschungsthemen wie genomweitem Assoziationsstudien
- ☐ Interesse an kommerziellen Gentests („Direct-to-Consumer Testing“)
- ☐ Um die Situation von Patienten bei genetischen Analysen besser nachvollziehen zu können
- ☐ Interesse an den ethischen Fragestellungen im Kontext von Genomanalysen
- ☐ Interesse an den rechtlichen Grundlagen von genetischen Analysen

---

Seite 05

4. Haben Sie sich im Rahmen des Kurses genotypisieren lassen?

- ○ Ja
- ○ Nein

Seite 06

5. Hat der Kurs Ihre Einstellungen gegenüber persönlichen Genotypanalysen verändert?

- ○ Ja
- ○ Nein

- ○ Ich weiß nicht

Seite 07

6. Wie hat der Kurs Ihre Einstellung verändert?

- ○ Durch den Kurs habe ich eine positivere Meinung zu genetischen Analysen gewonnen
- ○ Durch den Kurs bin ich kritischer in Bezug auf genetische Analysen geworden

Seite 08

7. Hätten Sie sich im Rahmen des Kurses genotypisieren lassen, wenn die Genotypisierung über einen nichteuropäischen kommerziellen Anbieter wie 23andMe durchgeführt worden wäre?

- Ja
- Nein

Seite 09

8. Haben Sie das Passwort für Ihre vollständigen Genotypdaten angefordert?

- Ja
- Nein

Seite 10

9. Haben Sie außerhalb des Kurses Analysen mit ihren Daten durchgeführt?

- Ja
- Nein

Seite 11

10. Planen Sie noch weitere Analysen mit Ihren Daten durchzuführen?

- Ja
- Nein

Seite 12

11. Würden Sie Verwandten oder Freunden, die den Kurs nicht belegt haben, eine persönliche Genotypisierung empfehlen?

- Ja
- Nein

Seite 13

12. Denken Sie, dass die Genotypisierung im Rahmen des Kurses nützlich für die Lernerfahrung ist?

- Ja
- Nein

Seite 14

13. Für wie wichtig halten Sie eine Behandlung von ethischen Aspekten im Kurs Genomische Medizin?

- Absolut notwendig
- Eher wichtig
- Neutral
- Eher unwichtig
- Völlig unwichtig

Seite 15

14. Würden Sie für die kommenden Kurse empfehlen, weiterhin eine Genotypisierung anzubieten?

- Ja
- Nein

Seite 16

15. Sollte die persönliche Genotypisierung ohne ärztliche genetische Beratung erlaubt sein?

- Ja
- Nein

Seite 17

16. Würden Sie es für sinnvoll halten, wenn ein vergleichbarer Kurs im Medizinstudium an Universitäten verpflichtend eingeführt würde?

- Ja
- Nein

Letzte Seite

Vielen Dank für Ihre Zeit

Ihre Antworten wurden abgeschickt, Sie können das Browser-Fenster oder den Tab jetzt schließen.

TUM Q4‘
(revised automatic English translation)

Page 01

Genomic medicine

Retrospective questionnaire

The purpose of this survey is to retrospectively assess the attitudes of participants in the *Genomic Medicine* course at the Technical University of Munich towards genome analyses. Similar surveys are being conducted in the currently running course and in the *Analyze Your Personal Genome* course at the Hasso Plattner Institute in Potsdam. This questionnaire takes about 5 minutes to complete.

Participation in this questionnaire is anonymous. We do not store any personal data.

Participation in this questionnaire is voluntary. You may choose not to participate.

We plan to publish the results of this survey anonymously. We will only use aggregated data for scientific publication, which will not allow any conclusions about the participants.

By continuing with the questionnaire, you are agreeing to the above terms.

- ○ I would like to participate in the survey
- ○ I do not wish to participate in the survey and would like to end the survey

---

Page 02

1. When did you participate in the course?

[Please select]

Options: SoSe 2017, WiSe 2017/18, SoSe 2018, WiSe 2018/19, WiSe 2019/20

Page 03

2. What did you study or what education did you have when you took the course?

[Please select]

Options: Medicine, Genetic and Genomic Counseling, Other educational background

3. What motivated you to attend the course?

- ☐ General interest in the topic of genetics/genome analyses.
- ☐ To obtain/analyze my own genetic data
- ☐ To gain knowledge about genetics/genome analyses and to use it in my profession
- ☐ To get to know tools that I can use to interpret and analyze genetic data
- ☐ Interest in genetic ancestry analyses
- ☐ Interest in pharmacogenomics (e.g., the effects of genetic variants on drug efficacy)
- ☐ Interest in research topics such as genome-wide association studies
- ☐ Interest in commercial genetic testing ("direct-to-consumer testing")
- ☐ To better understand the situation of patients in the context of genetic analyses
- ☐ Interest in the ethical issues in the context of genetic analyses
- ☐ Interest in the legal basis of genetic analyses

4. Did you opt in to get genotyped during the course?

- ○ Yes
- ○ No

5. Did the course change your attitudes toward personal genotype analyses?

- ○ Yes
- ○ No

- ○ I don’t know

6. How did the course change your attitude?

- ○ Because of the course I have gained a more positive opinion about genetic analyses
- ○ Because of the course I have become more critical of genetic analyses

Page 08

7. Would you have been genotyped as part of the course if the genotyping had been done through a non-European commercial provider such as 23andMe?

- Yes
- No

Page 09

8. Have you requested the password for your full genotype data?

- Yes
- No

Page 10

9. Have you done any analysis with your data outside of the course?

- Yes
- No

Page 11

10. Do you plan to do any further analysis with your data?

- Yes
- No

Page 12

11. Would you recommend personal genotyping to relatives or friends who have not taken the course?

- Yes
- No

Page 13

12. Do you think genotyping as part of the course is useful for the learning experience?

- Yes
- No

Page 14

13. How important do you think is it to address ethical issues in the Genomic Medicine course?

- Absolutely necessary
- Rather important
- Neutral
- Rather unimportant
- Completely unimportant

Page 15

14. For upcoming courses, would you recommend continuing to offer genotyping?

- Yes
- No

Page 16

15. Should personal genotyping be allowed without genetic counseling by a medical professional?

- Yes
- No

Page 17

16. Would you consider it useful if a comparable course was introduced as compulsory in the medical curriculum of universities?

- Yes
- No

Last page

Thank you for your time

Your answers have been submitted, you can close the browser window or tab now.

TUM 2020/21 Q1

Seite 01

Genomische Medizin

Fragebogen zu Kursbeginn

Das Ziel dieser Umfrage ist es, die Einstellung von Studierenden gegenüber Genomanalysen über den Kursverlauf zu erfassen. Die Umfrage wird in den Kursen *Genomische Medizin* an der Technischen Universität München und *Analyze Your Personal Genome* am Hasso-Plattner-Institut in Potsdam durchgeführt.

Insgesamt werden wir Sie bitten, vier Fragebögen zu beantworten: am Anfang des Kurses, während des Kurses, am Ende des Kurses und drei Monate nach dem Kurs.

Die Teilnahme an diesem Fragebogen ist freiwillig. Sie können sich dafür entscheiden, nicht teilzunehmen. Die Teilnahme an der Umfrage wird weder Ihre Note noch jeglichen anderen Aspekt Ihrer Teilnahme im Kurs oder Ihrer Ausbildung an der Technischen Universität München beeinflussen.

Die Teilnahme an diesem Fragebogen ist anonym. Wir speichern keine persönlichen Daten.

Wir planen, die Ergebnisse dieser Umfrage zu publizieren. Wir werden für die wissenschaftliche Veröffentlichung nur aggregierte Daten verwenden.

Wenn Sie mit dem Fragebogen fortfahren, stimmen Sie damit den oben genannten Bedingungen zu.

- ○ Ich möchte an der Umfrage teilnehmen
- ○ Ich möchte nicht an der Umfrage teilnehmen und möchte die Umfrage beenden

Seite 02
02

1. Was hat Sie motiviert, den Kurs zu belegen?

- ☐ Allgemeines Interesse am Thema Genetik/Genomanalysen
- ☐ Um meine eigenen genetischen Daten zu erhalten/analysieren
- ☐ Anrechnung des Wahlpflichtfachs
- ☐ Um Werkzeuge kennenzulernen, mit denen ich genetische Daten interpretieren und analysieren kann
- ☐ Interesse an genetischen Abstammungsanalysen
- ☐ Interesse an Pharmakogenomik (z.B. der Auswirkung von genetischen Varianten auf die Medikamentenwirkung)
- ☐ Interesse an Forschungsthemen wie genomweitem Assoziationsstudien
- ☐ Interesse an kommerziellen Gentests („Direct-to-Consumer Testing“)
- ☐ Um die Situation von Patienten bei genetischen Analysen besser nachvollziehen zu können
- ☐ Interesse an den ethischen Fragestellungen im Kontext von Genomanalysen
- ☐ Interesse an den rechtlichen Grundlagen von genetischen Analysen

---

Seite 03
03

2. Möchten Sie sich im Rahmen des Kurses genotypisieren lassen?

- ○ Ja
- ○ Nein

Seite 04
04

3. Planen Sie, nach dem Kurs das Passwort für Ihre vollständigen Genotypdaten anzufordern?

- ○ Ja
- ○ Nein

Seite 05
05

4. Welche Analysen planen Sie mit Ihren Daten durchzuführen?

- ☐ Keine, ich möchte einfach nur meine Daten bekommen
- ☐ Genetische Abstammungsanalysen
- ☐ Bestimmung pharmakogenomischer Varianten (Medikamentenwirkung)
- ☐ Bestimmung von nicht direkt krankheitsrelevanten genetischen Veranlagungen (z.B. Augenfarbe oder Blutgruppe)
- ☐ Diagnose von monogenen Krankheiten
- ☐ Trägerschaft von Risikovarianten für monogenetische Krankheiten bestimmen
- ☐ Risikoeinschätzung bei (häufigen) polygenen Krankheiten

Page 06
06

5. Sollte die persönliche Genotypisierung ohne ärztliche genetische Beratung erlaubt sein?

- ○ Ja
- ○ Nein

Last Page

Vielen Dank für Ihre Zeit

Ihre Antworten wurden abgeschickt, Sie können das Browser-Fenster oder den Tab jetzt schließen.

TUM 2020/21 Q1
(revised automatic English translation)

Genomic Medicine

Questionnaire at the beginning of the course

The purpose of this survey is to assess the attitudes of students towards genome analyses during the course. The survey is conducted in the courses *Genomic Medicine* at the Technical University of Munich and *Analyze Your Personal Genome* at the Hasso Plattner Institute in Potsdam.

In total, we will ask you to participate in four questionnaires: at the beginning of the course, during the course, at the end of the course, and three months after the course.

Participation in this questionnaire is voluntary. You may choose not to participate. Participation in the survey will not affect your grade or any other aspect regarding your participation in the course or your education at the Technical University of Munich.

Participation in this questionnaire is anonymous. We do not store any personal data.

We plan to publish the results of this survey. We will only use aggregated data for scientific publication.

By continuing with the questionnaire, you are agreeing to the above terms.

- ○ I would like to participate in the survey
- ○ I do not wish to participate in the survey and would like to end the survey

Page 02
02

1. What motivated you to attend the course?

- ☐ General interest in the subject of genetics/genome analyses.
- ☐ To obtain/analyze my own genetic data.
- ☐ To receive the credit for my studies
- ☐ To get to know tools that I can use to interpret and analyze genetic data
- ☐ Interest in genetic ancestry analysis
- ☐ Interest in pharmacogenomics (e.g., the effects of genetic variants on drug efficacy)
- ☐ Interest in research topics such as genome-wide association studies
- ☐ Interest in commercial genetic testing ("direct-to-consumer testing").
- ☐ To better understand the situation of patients in the context of genetic analyses
- ☐ Interest in the ethical issues in the context of genomic analyses
- ☐ Interest in the legal basis of genetic analyses

Page 03
03

2. Would you like to get genotyped during the course?

- ○ Yes
- ○ No

Page 04
04

3. Do you plan to request the password for your full genotype data after the course?

- ○ Yes
- ○ No

Page 05
05

4. Which analyses do you plan to conduct with your data?

- ☐ None, I just want to receive my data
- ☐ Genetic ancestry analyses
- ☐ Assessment of pharmacogenomic variants (drug effects)
- ☐ Assessment of genetic predispositions not directly relevant to disease (e.g., eye color or blood type)
- ☐ Diagnosis of monogenic diseases
- ☐ Assessment of carrier status for risk variants of monogenetic diseases
- ☐ Risk assessment for (common) polygenic diseases

Page 06
06

5. Should personal genotyping be allowed without genetic counseling by a medical professional?

- ○ Yes
- ○ No

Last Page

Thank you for your time

Your answers have been submitted, you can close the browser window or tab now.

TUM 2020/21 Q2

Seite 01

Genomische Medizin

Fragebogen während des Kurses

Das Ziel dieser Umfrage ist es, die Einstellung von Studierenden gegenüber Genomanalysen über den Verlauf des Wahlfaches hinweg zu erfassen. Die Umfrage wird in den Kursen *Genomische Medizin* an der Technischen Universität München und *Analyze Your Personal Genome* am Hasso-Plattner-Institut in Potsdam durchgeführt. Die Beantwortung dieses Fragebogens dauert etwa 5 Minuten.

Insgesamt besteht die Umfrage aus vier Fragebögen: am Anfang des Kurses, während des Kurses, am Ende des Kurses und drei Monate nach dem Kurs.

Die Teilnahme an diesem Fragebogen ist anonym. Wir speichern keine persönlichen Daten.

Die Teilnahme an diesem Fragebogen ist freiwillig. Sie können sich dafür entscheiden, nicht teilzunehmen. Die Teilnahme an der Umfrage wird weder Ihre Note noch Ihre Teilnahme am Wahlfach beeinflussen.

Wir planen, die Ergebnisse dieser Umfrage anonymisiert zu publizieren. Wir werden für die wissenschaftliche Veröffentlichung nur aggregierte Daten verwenden, die keine Rückschlüsse auf die Teilnehmer erlauben.

Wenn Sie mit dem Fragebogen fortfahren, stimmen Sie den oben genannten Bedingungen zu.

- ○ Ich möchte an der Umfrage teilnehmen
- ○ Ich möchte nicht an der Umfrage teilnehmen und möchte die Umfrage beenden

---

Seite 02

1. Haben Sie sich im Rahmen des Kurses genotypisieren lassen?

- ○ Ja
- ○ Nein

Seite 03

2. Würden Sie sich aus heutiger Sicht wieder genotypisieren lassen?

- ○ Ja
- ○ Nein

3. Würden Sie sich aus heutiger Sicht genotypisieren lassen?

- Ja
- Nein

Seite 04

4. Hätten Sie sich im Rahmen des Kurses genotypisieren lassen, wenn die Genotypisierung über einen nichteuropäischen kommerziellen Anbieter wie 23andMe durchgeführt worden wäre?

- Ja
- Nein

Seite 05

5. Planen Sie, nach dem Kurs das Passwort für Ihre vollständigen Genotypdaten anzufordern?

- Ja
- Nein

Seite 06

6. Welche Analysen würden Sie mit Ihren eigenen Daten durchführen?

- Genetische Abstammungsanalysen
- Bestimmung pharmakogenomischer Varianten (Medikamentenwirkung)
- Bestimmung von nicht direkt krankheitsrelevanten genetischen Veranlagungen (z.B. Augenfarbe oder Blutgruppe)
- Trägerschaft von Risikovarianten für monogene Krankheiten bestimmen
- Risikoeinschätzung bei (häufigen) polygenen Krankheiten
- Keine, ich wollte einfach nur meine Daten bekommen

Seite 07

7. Sollte die persönliche Genotypisierung ohne ärztliche genetische Beratung erlaubt sein?

- Ja
- Nein

Seite 08

8. Würden Sie Verwandten oder Freunden, die den Kurs nicht belegt haben, eine persönliche Genotypisierung empfehlen?

- Ja
- Nein

Seite 09

9. Für wie wichtig halten Sie eine Behandlung von ethischen Aspekten im Kurs Genomische Medizin?

- Absolut notwendig
- Eher wichtig
- Neutral
- Eher unwichtig
- Völlig unwichtig

Seite 10

10. Würden Sie es für sinnvoll halten, wenn ein vergleichbarer Kurs im Medizinstudium an Universitäten verpflichtend eingeführt würde?

- Ja
- Nein

Letzte Seite

Vielen Dank für Ihre Zeit

Ihre Antworten wurden gespeichert, Sie können das Browser-Fenster oder den Tab jetzt schließen.

TUM 2020/21 Q2 (revised automatic English translation)

Page 01

Genomic Medicine

Questionnaire during the course

The purpose of this survey is to assess the attitudes of students towards genome analyses during the course. The survey is conducted in the courses *Genomic Medicine* at the Technical University of Munich and *Analyze Your Personal Genome* at the Hasso Plattner Institute in Potsdam. This questionnaire takes about 5 minutes to complete.

In total, we will ask you to participate in four questionnaires: at the beginning of the course, during the course, at the end of the course, and three months after the course.

Participation in this questionnaire is anonymous. We do not store any personal data.

Participation in this questionnaire is voluntary. You may choose not to participate. Participation in the survey will not affect your grade or your participation in the course.

We plan to publish the results of this survey anonymously. We will only use aggregated data for scientific publication, which will not allow any conclusions about the participants.

By continuing with the questionnaire, you are agreeing to the above terms.

- ○ I would like to participate in the survey
- ○ I do not wish to participate in the survey and would like to end the survey

Page 02

1. Did you opt in to get genotyped during the course?

- ○ Yes
- ○ No

Page 03

2. From today's perspective, would you have opted in for the genotyping again?

- ○ Yes
- ○ No

3. From today's perspective, would you have opted in for the genotyping?

- Yes
- No

Page 04

4. Would you have been genotyped as part of the course if the genotyping had been done through a non-European commercial provider such as 23andMe?

- Yes
- No

Page 05

5. Do you plan to request the password for your full genotype data after the course?

- Yes
- No

Page 06

6. Which analyses would you conduct with your data?

- Genetic ancestry analyses
- Assessment of pharmacogenomic variants (drug effects)
- Assessment of genetic predispositions not directly relevant to disease (e.g., eye color or blood type)
- Assessment of carrier status for risk variants of monogenic diseases
- Risk assessment for (common) polygenic diseases
- None, I just wanted to receive my data

Page 07

7. Should personal genotyping be allowed without genetic counseling by a medical professional?

- Yes
- No

Page 08

8. Would you recommend personal genotyping to relatives or friends who have not taken the course?

- Yes
- No

Page 09

9. How important do you think is it to address ethical issues in the Genomic Medicine course?

- Absolutely necessary
- Rather important
- Neutral
- Rather unimportant
- Completely unimportant

Page 10

10. Would you consider it useful if a comparable course was introduced as compulsory in the medical curriculum of universities?

- Yes
- No

Last page

Thank you for your time.

Your answers have been saved, you can close the browser window or tab now.

TUM 2020/21 Q3

Seite 01

Genomische Medizin

Fragebogen zu Kursende

Das Ziel dieser Umfrage ist es, die Einstellung von Studierenden gegenüber Genomanalysen über den Verlauf des Wahlfaches hinweg zu erfassen. Die Umfrage wird in den Kursen *Genomische Medizin* an der Technischen Universität München und *Analyze Your Personal Genome* am Hasso-Plattner-Institut in Potsdam durchgeführt. Die Beantwortung dieses Fragebogens dauert etwa 5 Minuten.

Insgesamt besteht die Umfrage aus vier Fragebögen: am Anfang des Kurses, während des Kurses, am Ende des Kurses und drei Monate nach dem Kurs.

Die Teilnahme an diesem Fragebogen ist anonym. Wir speichern keine persönlichen Daten.

Die Teilnahme an diesem Fragebogen ist freiwillig. Sie können sich dafür entscheiden, nicht teilzunehmen. Die Teilnahme an der Umfrage wird weder Ihre Note noch Ihre Teilnahme am Wahlfach beeinflussen.

Wir planen, die Ergebnisse dieser Umfrage anonymisiert zu publizieren. Wir werden für die wissenschaftliche Veröffentlichung nur aggregierte Daten verwenden, die keine Rückschlüsse auf die Teilnehmer erlauben.

Wenn Sie mit dem Fragebogen fortfahren, stimmen Sie den oben genannten Bedingungen zu.

- ○ Ich möchte an der Umfrage teilnehmen
- ○ Ich möchte nicht an der Umfrage teilnehmen und möchte die Umfrage beenden

---

Seite 02

1. Haben Sie sich im Rahmen des Kurses genotypisieren lassen?

- ○ Ja
- ○ Nein

Seite 03

2. Würden Sie sich aus heutiger Sicht wieder genotypisieren lassen?

- ○ Ja
- ○ Nein

3. Würden Sie sich aus heutiger Sicht genotypisieren lassen?

- Ja
- Nein

Seite 04

4. Hat der Kurs Ihre Einstellungen gegenüber persönlichen Genotypanalysen verändert?

- Ja
- Nein

- Ich weiß nicht

Seite 05

5. Wie hat der Kurs Ihre Einstellung verändert?

- Durch den Kurs habe ich eine positivere Meinung zu genetischen Analysen gewonnen
- Durch den Kurs bin ich kritischer in Bezug auf genetische Analysen geworden

---

Seite 06

6. Hätten Sie sich im Rahmen des Kurses genotypisieren lassen, wenn die Genotypisierung über einen nichteuropäischen kommerziellen Anbieter wie 23andMe durchgeführt worden wäre?

- Ja
- Nein

Seite 07

7. Planen Sie, nach dem Kurs das Passwort für Ihre vollständigen Genotypdaten anzufordern?

- Ja
- Nein

Seite 08

8. Welche Analysen würden Sie mit Ihren eigenen Daten durchführen?

- ☐ Genetische Abstammungsanalysen
- ☐ Bestimmung pharmakogenomischer Varianten (Medikamentenwirkung)
- ☐ Bestimmung von nicht direkt krankheitsrelevanten genetischen Veranlagungen (z.B. Augenfarbe oder Blutgruppe)
- ☐ Trägerschaft von Risikovarianten für monogene Krankheiten bestimmen
- ☐ Risikoeinschätzung bei (häufigen) polygenen Krankheiten
- ☐ Keine, ich wollte einfach nur meine Daten bekommen

Seite 09

9. Würden Sie Verwandten oder Freunden, die den Kurs nicht belegt haben, eine persönliche Genotypisierung empfehlen?

- ○ Ja
- ○ Nein

Seite 10

10. Denken Sie, dass die Genotypisierung im Rahmen des Kurses nützlich für die Lernerfahrung ist?

- ○ Ja
- ○ Nein

Seite 11

11. Für wie wichtig halten Sie eine Behandlung von ethischen Aspekten im Kurs Genomische Medizin?

- ○ Absolut notwendig
- ○ Eher wichtig
- ○ Neutral
- ○ Eher unwichtig
- ○ Völlig unwichtig

Seite 12

12. Würden Sie für die kommenden Kurse empfehlen, weiterhin eine Genotypisierung anzubieten?

- ○ Ja
- ○ Nein

Seite 13

13. Sollte die persönliche Genotypisierung ohne ärztliche genetische Beratung erlaubt sein?

- Ja
- Nein

Seite 14

14. Würden Sie es für sinnvoll halten, wenn ein vergleichbarer Kurs im Medizinstudium an Universitäten verpflichtend eingeführt würde?

- Ja
- Nein

Letzte Seite

Vielen Dank für Ihre Zeit

Ihre Antworten wurden gespeichert, Sie können das Browser-Fenster oder den Tab jetzt schließen.

TUM 2020/21 Q3
(revised automatic English translation)

Page 01

Genomic Medicine

Questionnaire at the end of the course

The purpose of this survey is to assess the attitudes of students towards genome analyses during the course. The survey is conducted in the courses *Genomic Medicine* at the Technical University of Munich and *Analyze Your Personal Genome* at the Hasso Plattner Institute in Potsdam. This questionnaire takes about 5 minutes to complete.

In total, we will ask you to participate in four questionnaires: at the beginning of the course, during the course, at the end of the course, and three months after the course.

Participation in this questionnaire is anonymous. We do not store any personal data.

Participation in this questionnaire is voluntary. You may choose not to participate. Participation in the survey will not affect your grade or your participation in the course.

We plan to publish the results of this survey anonymously. We will only use aggregated data for scientific publication, which will not allow any conclusions about the participants.

By continuing with the questionnaire, you are agreeing to the above terms.

- ○ I would like to participate in the survey
- ○ I do not wish to participate in the survey and would like to end the survey

---

Page 02

1. Did you opt in to get genotyped during the course?

- ○ Yes
- ○ No

Page 03

2. From today's perspective, would you have opted in for the genotyping again?

- ○ Yes
- ○ No

3. From today's perspective, would you have opted in for the genotyping?

- Yes
- No

Page 04

4. Did the course change your attitudes toward personal genotype analysis?

- Yes
- No

- I don’t know

Page 05

5. How did the course change your attitude?

- Because of the course I have gained a more positive opinion about genetic analyses
- Because of the course I have become more critical about genetic analyses

---

Page 06

6. Would you have been genotyped as part of the course if the genotyping had been done through a non-European commercial provider such as 23andMe?

- Yes
- No

Page 07

7. Do you plan to request the password for your full genotype data after the course?

- Yes
- No

Page 08

8. Which analyses would you conduct with your data?

- ☐ Genetic ancestry analyses
- ☐ Assessment of pharmacogenomic variants (drug effects)
- ☐ Assessment of genetic predispositions not directly relevant to disease (e.g., eye color or blood type)
- ☐ Assessment of carrier status for risk variants of monogenic diseases
- ☐ Risk assessment for (common) polygenic diseases
- ☐ None, I just wanted to receive my data

Page 09

9. Would you recommend personal genotyping to relatives or friends who have not taken the course?

- ○ Yes
- ○ No

Page 10

10. Do you think genotyping as part of the course is useful for the learning experience?

- ○ Yes
- ○ No

Page 11

11. How important do you think is it to address ethical issues in the Genomic Medicine course?

- ○ Absolutely necessary
- ○ Rather important
- ○ Neutral
- ○ Rather unimportant
- ○ Completely unimportant

Page 12

12. Would you recommend continuing to offer genotyping for upcoming courses?

- ○ Yes
- ○ No

Page 13

13. Should personal genotyping be allowed without genetic counseling by a medical professional?

- Yes
- No

Seite 14

14. Would you consider it useful if a comparable course was introduced as compulsory in the medical curriculum of universities?

- Yes
- No

Last page

Thank you for your time.

Your answers have been saved, you can close the browser window or tab now.

TUM 2020/21 Q4

Seite 01

Retrospektiver Fragebogen

Genomische Medizin

Das Ziel dieser Umfrage ist es, die Einstellung von Studierenden gegenüber Genomanalysen über den Kursverlauf zu erfassen. Die Umfrage wird in den Kursen *Genomische Medizin* an der Technischen Universität München und *Analyze Your Personal Genome* am Hasso-Plattner-Institut in Potsdam durchgeführt.

Insgesamt werden wir Sie bitten, vier Fragebögen zu beantworten: am Anfang des Kurses, während des Kurses, am Ende des Kurses und drei Monate nach dem Kurs.

Die Teilnahme an diesem Fragebogen ist freiwillig. Sie können sich dafür entscheiden, nicht teilzunehmen. Die Teilnahme an der Umfrage wird weder Ihre Note noch jeglichen anderen Aspekt Ihrer Teilnahme im Kurs oder Ihrer Ausbildung an der Technischen Universität München beeinflussen.

Die Teilnahme an diesem Fragebogen ist anonym. Wir speichern keine persönlichen Daten.

Wir planen, die Ergebnisse dieser Umfrage zu publizieren. Wir werden für die wissenschaftliche Veröffentlichung nur aggregierte Daten verwenden.

Wenn Sie mit dem Fragebogen fortfahren, stimmen Sie damit den oben genannten Bedingungen zu.

- ○ Ich möchte an der Umfrage teilnehmen
- ○ Ich möchte nicht an der Umfrage teilnehmen und möchte die Umfrage beenden

---

Seite 02

1. Haben Sie sich im Rahmen des Kurses genotypisieren lassen?

- ○ Ja
- ○ Nein

Seite 03

2. Würden Sie sich aus heutiger Sicht genotypisieren lassen?

- ○ Ja
- ○ Nein

3. Würden Sie sich aus heutiger Sicht wieder genotypisieren lassen?

- ○ Ja
- ○ Nein

Seite 04

4. Hat der Kurs Ihre Einstellungen gegenüber persönlichen Genotypanalysen verändert?

- ◯ Ja
- ◯ Nein
- ◯ Ich weiß nicht

Seite 05

5. Wie hat der Kurs Ihre Einstellung verändert?

- ◯ Durch den Kurs habe ich eine positivere Meinung zu genetischen Analysen gewonnen
- ◯ Durch den Kurs bin ich kritischer in Bezug auf genetische Analysen geworden

Seite 06

6. Würden Sie sich im Rahmen des Kurses genotypisieren lassen, wenn die Genotypisierung über einen nichteuropäischen kommerziellen Anbieter wie 23andMe durchgeführt werden würde?

- ◯ Ja
- ◯ Nein

Seite 07

7. Haben Sie das Passwort für Ihre vollständigen Genotypdaten angefordert?

- ◯ Ja
- ◯ Nein

Seite 08

8. Planen Sie noch, das Passwort für Ihre vollständigen Genotypdaten anzufordern?

- ◯ Ja
- ◯ Nein

Seite 09

9. Haben Sie außerhalb des Kurses Analysen mit Ihren Daten durchgeführt?

- ☐ Genetische Abstammungsanalysen
- ☐ Bestimmung pharmakogenomischer Varianten (Medikamentenwirkung)
- ☐ Bestimmung von nicht direkt krankheitsrelevanten genetischen Veranlagungen (z.B. Augenfarbe oder Blutgruppe)
- ☐ Trägerschaft von Risikovarianten für monogene Krankheiten bestimmen
- ☐ Risikoeinschätzung bei (häufigen) polygenen Krankheiten
- ☐ Nein, ich wollte einfach nur meine Daten bekommen

Seite 10

10. Planen Sie noch weitere Analysen mit Ihren Daten durchzuführen?

- ○ Ja
- ○ Nein

Seite 11

11. Sollte die persönliche Genotypisierung ohne ärztliche genetische Beratung erlaubt sein?

- ○ Ja
- ○ Nein

Seite 12

12. Würden Sie Verwandten oder Freunden, die den Kurs nicht belegt haben, eine persönliche Genotypisierung empfehlen?

- ○ Ja
- ○ Nein

Seite 13

13. Für wie wichtig halten Sie eine Behandlung von ethischen Aspekten im Kurs Genomische Medizin?

- ○ Absolut notwendig
- ○ Eher wichtig
- ○ Neutral
- ○ Eher unwichtig
- ○ Völlig unwichtig

Seite 14

14. Denken Sie, dass die Genotypisierung im Rahmen des Kurses nützlich für die Lernerfahrung ist?

- Ja
- Nein

Seite 15

15. Würden Sie für die kommenden Kurse empfehlen, weiterhin eine Genotypisierung anzubieten?

- Ja
- Nein

Seite 16

16. Würden Sie es für sinnvoll halten, wenn ein vergleichbarer Kurs im Medizinstudium an Universitäten verpflichtend eingeführt würde?

- Ja
- Nein

Seite 17

17. Fühlen Sie sich ausreichend ausgebildet, um Genotypdaten über den Kurs hinaus zu analysieren und zu interpretieren?

- Ja
- Nein

Seite 18

18. Wenn Sie diesen Kurs nochmals belegen könnten, was würden Sie anders machen und warum?

Seite 19

19. Vielen Dank, dass Sie uns helfen, die Vorlesung zu verbessern und zu erfahren, wie Studierende den Thema Genomanalyse gegenüberstehen. Wenn Sie weitere Anmerkungen haben, hinterlassen Sie diese bitte hier:

Vielen Dank für Ihre Zeit

Ihre Antworten wurden gespeichert, Sie können das Browser-Fenster oder den Tab jetzt schließen.

TUM 2020/21 Q4
(revised automatic English translation)

Page 01

Retrospective Questionnaire

Genomic Medicine

The purpose of this survey is to assess the attitudes of students towards genome analyses during the course. The survey is conducted in the courses *Genomic Medicine* at the Technical University of Munich and *Analyze Your Personal Genome* at the Hasso Plattner Institute in Potsdam.

In total, we will ask you to participate in four questionnaires: at the beginning of the course, during the course, at the end of the course, and three months after the course.

Participation in this questionnaire is voluntary. You may choose not to participate. Participation in the survey will not affect your grade or any other aspect regarding your participation in the course or your education at the Technical University of Munich.

Participation in this questionnaire is anonymous. We do not store any personal data.

We plan to publish the results of this survey. We will only use aggregated data for scientific publication.

By continuing with the questionnaire, you are agreeing to the above terms.

- ◯ I would like to participate in the survey
- ◯ I do not wish to participate in the survey and would like to end the survey

---

Page 02

1. Did you opt in to get genotyped during the course?

- ◯ Yes
- ◯ No

Page 03

2. From today's perspective, would you have opted in for the genotyping again?

- ◯ Yes
- ◯ No

3. From today's perspective, would you have opted in for the genotyping?

- ◯ Yes
- ◯ No

Page 04

4. Did the course change your attitudes toward personal genotype analysis?

- Yes
- No
- I don’t know

Page 05

5. How did the course change your attitude?

- Because of the course I have gained a more positive opinion about genetic analyses
- Because of course I have become more critical about genetic analyses

Page 06

6. Would you have been genotyped as part of the course if the genotyping had been done through a non-European commercial provider such as 23andMe?

- Yes
- No

Page 07

7. Have you requested the password for your full genotype data?

- Yes
- No

Page 08

8. Are you still planning to request the password for your full genotype data?

- Yes
- No

Page 09

9. Have you conducted any analyses with your data outside of the course?

- ☐ Genetic ancestry analyses
- ☐ Assessment of pharmacogenomic variants (drug effects)
- ☐ Assessment of genetic predispositions not directly relevant to disease (e.g., eye color or blood type)
- ☐ Assessment of carrier status for risk variants of monogenic diseases
- ☐ Risk assessment for (common) polygenic diseases
- ☐ None, I just wanted to receive my data

Page 10

10. Do you plan to conduct any further analyses with your data?

- ○ Yes
- ○ No

Page 11

11. Should personal genotyping be allowed without genetic counseling by a medical professional?

- ○ Yes
- ○ No

Page 12

12. Would you recommend personal genotyping to relatives or friends who have not taken the course?

- ○ Yes
- ○ No

Page 13

13. How important do you think is it to address ethical issues in the Genomic Medicine course?

- ○ Absolutely necessary
- ○ Rather important
- ○ Neutral
- ○ Rather unimportant
- ○ Completely unimportant

Page 14

14. Do you think genotyping as part of the course is useful for the learning experience?

- ○ Yes
- ○ No

Page 15

15. For upcoming courses, would you recommend continuing to offer genotyping?

- ○ Yes
- ○ No

Page 16

16. Would you consider it useful if a comparable course was introduced as compulsory in the medical curriculum of universities?

- ○ Yes
- ○ No

Page 17

17. Do you feel you have sufficient training to analyze and interpret genotype data beyond the course?

- ○ Yes
- ○ No

Page 18

18. If you could take this course again, what would you do differently and why?

Page 19

19. Thank you for helping us to improve the lecture and to learn about the students' attitude towards the topic of genome analysis. If you have any further comments, please leave them here:

Thank you for your time

Your answers have been saved, you can close the browser window or tab now.

HPI 2021 Q1

Page 01

Pre-Course Questionnaire

Understanding Your Genome

The purpose of this questionnaire is to compare students' attitudes regarding personal genotyping over the course. The survey is conducted in the courses *Genomische Medizin* at the Technical University of Munich and *Understanding Your Genome* at Hasso Plattner Institute in Potsdam. Filling out this questionnaire will take less than 5 minutes.

In total, this survey consists of four questionnaires: before the course, during the course, shortly after the course, and three months after the course.

Your participation in this questionnaire is voluntary. You may choose not to participate. If you choose to participate, you may stop at any time without any penalty. This will not affect your participation, grade, or any other aspect of your involvement in the course, or any other aspect of your education at HPI.

Your participation in this questionnaire is anonymous to the teaching staff. Your name or other information that could identify you will not be on the questionnaires. Your responses will only be identified by a pseudonym so that we can follow the development of answers throughout questionnaires. Only the course honest broker knows the mapping of pseudonyms and names and has no access to the pseudonymized results. We will anonymize the data by deleting the mapping and additionally assigning new, random pseudonyms.

We plan to publish the results of this survey in a scientific journal. We will only use anonymous data for the publication and we will not cite free text answers.

By continuing with the questionnaire, you confirm that you have read and understood the information above and agree to the conditions for participation.

- ○ I want to participate in the study
- ○ I do <u>not</u> want to participate in the study and want to exit

Page 02

1. Did you study medicine?

- ○ Yes
- ○ No

Page 03
02

2. What motivated you to participate in the course?

- General interest in genomics/genomic analyses
- To receive/analyze my own genomic data
- To gain knowledge about genomics/genomic analyses to apply it in my professional career
- To receive the credit points
- To learn about tools for variant interpretation and analysis
- Interest in ancestry analysis
- Interest in pharmacogenomics (e.g., the impact of genomic variants on medication effects)
- Interest in research topics like genome-wide association studies
- Interest in commercial genomic testing (“direct-to-consumer testing”)
- To better understand the situation of patients when undergoing genomic testing
- Interest in ethical issues in the context of genomic analyses
- Interest in legal foundations of genomic analyses

Page 04
03

3. Do you plan to get genotyped as part of the course?

- Yes
- No

Page 05

4. Would you participate in personal genotyping as part of the course if it would be offered via a non-european private company, such as 23andMe?

- Yes
- No

Page 06
04

5. Do you plan to request the password for your complete genomic data after the course?

- ○ Yes
- ○ No

Page 07

6. Which analyses do you plan to conduct with your own genomic data?

- ☐ Analyzing genetic ancestry
- ☐ Analyzing pharmacogenomic markers (medication effects)
- ☐ Analyzing wellness traits (not disease-relevant, e.g., eye color or blood type)
- ☐ Carrier detection of risk variants for monogenetic diseases
- ☐ Analyzing risk of (common) polygenic diseases
- ☐ None, I just want to receive my data

Page 08
06

7. Should personal genotyping be allowed without genetic counseling by a medical professional?

- ○ Yes
- ○ No

Page 09

8. Would you recommend personal genotyping to relatives or friends who not took the course?

- ○ Yes
- ○ No

9. How important do you consider the treatment of ethical aspects in the Understanding Your Genome course?

- Absolutely necessary
- Rather important
- Neutral
- Rather unimportant
- Completely unimportant

Thank you for your time!

Your answers were transmitted, you may close the browser window or tab now.

HPI 2021 Q2

Page 01

Intermediate Questionnaire

Understanding Your Genome

The purpose of this questionnaire is to compare students' attitudes regarding personal genotyping over the course. The survey is conducted in the courses *Genomische Medizin* at the Technical University of Munich and *Understanding Your Genome* at Hasso Plattner Institute in Potsdam. Filling out this questionnaire will take less than 5 minutes.

In total, this survey consists of four questionnaires: before the course, during the course, shortly after the course, and three months after the course.

Your participation in this questionnaire is voluntary. You may choose not to participate. If you choose to participate, you may stop at any time without any penalty. This will not affect your participation, grade, or any other aspect of your involvement in the course, or any other aspect of your education at HPI.

Your participation in this questionnaire is anonymous to the teaching staff. Your name or other information that could identify you will not be on the questionnaires. Your responses will only be identified by a pseudonym so that we can follow the development of answers throughout questionnaires. Only the course honest broker knows the mapping of pseudonyms and names and has no access to the pseudonymized results. We will anonymize the data by deleting the mapping and additionally assigning new, random pseudonyms.

We plan to publish the results of this survey in a scientific journal. We will only use anonymous data for the publication and we will not cite free text answers.

By continuing with the questionnaire, you confirm that you have read and understood the information above and agree to the conditions for participation.

- ○ I want to participate in the study
- ○ I do <u>not</u> want to participate in the study and want to exit

---

Page 02

1. Did you study medicine?

- ○ Yes
- ○ No

Page 03

2. Did you participate in personal genotyping as part of the course?

- ○ Yes
- ○ No

Page 04

3. From today's perspective, would you have yourself genotyped?

- ○ Yes
- ○ No

4. From today's perspective, would you have yourself genotyped again?

- ○ Yes
- ○ No

Page 05

5. Would you participate in personal genotyping as part of the course if it would be offered via a non-european private company, such as 23andMe?

- ○ Yes
- ○ No

Page 06

6. Do you plan to request your complete genomic data after the course?

- ○ Yes
- ○ No

Page 07

7. Which analyses do you plan to conduct with your own genomic data?

- ☐ Analyzing genetic ancestry
- ☐ Analyzing pharmacogenomic markers (medication effects)
- ☐ Analyzing wellness traits (not disease-relevant, e.g., eye color or blood type)
- ☐ Carrier detection of risk variants for monogenetic diseases
- ☐ Analyzing risk of (common) polygenic diseases
- ☐ None, I just want to receive my data

Page 08

8. Should personal genotyping be allowed without genetic counseling by a medical professional?

- ○ Yes
- ○ No

Page 09

9. Would you recommend personal genotyping to relatives or friends who did not take the course?

- ○ Yes
- ○ No

Page 10

10. How important do you consider the treatment of ethical aspects in the Understanding Your Genome course?

- ○ Absolutely necessary
- ○ Rather important
- ○ Neutral
- ○ Rather unimportant
- ○ Completely unimportant

Last Page

Thank you for your time!

Your answers were transmitted, you may close the browser window or tab now.

HPI 2021 Q3

Page 01

End-Course Questionnaire

Understanding Your Genome

The purpose of this questionnaire is to compare students' attitudes regarding personal genotyping over the course. The survey is conducted in the courses *Genomische Medizin* at the Technical University of Munich and *Understanding Your Genome* at Hasso Plattner Institute in Potsdam. Filling out this questionnaire will take less than 5 minutes.

In total, this survey consists of four questionnaires: before the course, during the course, shortly after the course, and three months after the course.

Your participation in this questionnaire is voluntary. You may choose not to participate. If you choose to participate, you may stop at any time without any penalty. This will not affect your participation, grade, or any other aspect of your involvement in the course, or any other aspect of your education at HPI.

Your participation in this questionnaire is anonymous to the teaching staff. Your name or other information that could identify you will not be on the questionnaires. Your responses will only be identified by a pseudonym so that we can follow the development of answers throughout questionnaires. Only the course honest broker knows the mapping of pseudonyms and names and has no access to the pseudonymized results. We will anonymize the data by deleting the mapping and additionally assigning new, random pseudonyms.

We plan to publish the results of this survey in a scientific journal. We will only use anonymous data for the publication and we will not cite free text answers.

By continuing with the questionnaire, you confirm that you have read and understood the information above and agree to the conditions for participation.

- ○ I want to participate in the study
- ○ I do <u>not</u> want to participate in the study and want to exit

---

Page 02

1. Did you study medicine?

- ○ Yes
- ○ No

Page 03

2. Did you participate in personal genotyping as part of the course?

- ○ Yes
- ○ No

Page 04

3. From today's perspective, would you have yourself genotyped?

- ◯ Yes
- ◯ No

4. From today's perspective, would you have yourself genotyped again?

- ◯ Yes
- ◯ No

Page 05

5. Has this course changed your attitude towards personal genotype analysis?

- ◯ Yes
- ◯ No

- ◯ I don't know

Page 06

6. How did the course change your attitude?

- ◯ Through the course I have gained a more positive opinion on genetic analysis
- ◯ Through the course I have become more critical about genetic analysis

Page 07

7. Would you participate in personal genotyping as part of the course if it would be offered via a non-european private company, such as 23andMe?

- ◯ Yes
- ◯ No

Page 08

8. Do you plan to request your complete genomic data after the course?

- ◯ Yes
- ◯ No

Page 09

9. Which analyses do you plan to conduct with your own genomic data?

- ☐ Analyzing genetic ancestry
- ☐ Analyzing pharmacogenomic markers (medication effects)
- ☐ Analyzing wellness traits (not disease-relevant, e.g., eye color or blood type)
- ☐ Carrier detection of risk variants for monogenetic diseases
- ☐ Analyzing risk of (common) polygenic diseases
- ☐ None, I just want to receive my data

Page 10

10. Should personal genotyping be allowed without genetic counseling by a medical professional?

- ○ Yes
- ○ No

Page 11

11. Would you recommend personal genotyping to relatives or friends who did not take the course?

- ○ Yes
- ○ No

Page 12

12. How important do you consider the treatment of ethical aspects in the Understanding Your Genome course?

- ○ Absolutely necessary
- ○ Rather important
- ○ Neutral
- ○ Rather unimportant
- ○ Completely unimportant

Page 13

13. Do you think that personal genotyping in the course context is useful for the learning experience?

- ○ Yes
- ○ No

Page 14

14. Would you recommend to offer personal genotyping in future courses again?

- ○ Yes
- ○ No

Page 15

15. Do you feel adequately trained to analyze and interpret genomic data beyond the course?

- ○ Yes
- ○ No

Page 16

16. What worked well in the course? Please tell us why.

Page 17

17. What did not work well in the course? Please tell us why.

Page 18

18. A big thank you for helping us to improve the lecture and to learn about your attitudes towards genome analysis. If you have any further comments, please leave them here:

Thank you for your time!

Your answers were transmitted, you may close the browser window or tab now.

HPI 2021 Q4

Page 01

Retrospective Questionnaire

Understanding Your Genome

The purpose of this questionnaire is to compare students' attitudes regarding personal genotyping over the course. The survey is conducted in the courses *Genomische Medizin* at the Technical University of Munich and *Understanding Your Genome* at Hasso Plattner Institute in Potsdam. Filling out this questionnaire will take less than 5 minutes.

In total, this survey consists of four questionnaires: before the course, during the course, shortly after the course, and three months after the course.

Your participation in this questionnaire is voluntary. You may choose not to participate. If you choose to participate, you may stop at any time without any penalty. This will not affect your participation, grade, or any other aspect of your involvement in the course, or any other aspect of your education at HPI.

Your participation in this questionnaire is anonymous to the teaching staff. Your name or other information that could identify you will not be on the questionnaires. Your responses will only be identified by a pseudonym so that we can follow the development of answers throughout questionnaires. Only the course honest broker knows the mapping of pseudonyms and names and has no access to the pseudonymized results. We will anonymize the data by deleting the mapping and additionally assigning new, random pseudonyms.

We plan to publish the results of this survey in a scientific journal. We will only use anonymous data for the publication and we will not cite free text answers.

By continuing with the questionnaire, you confirm that you have read and understood the information above and agree to the conditions for participation.

- ◯ I want to participate in the study
- ◯ I do <u>not</u> want to participate in the study and want to exit

---

Page 02

1. Did you study medicine?

- ◯ Yes
- ◯ No

Page 03

2. Did you participate in personal genotyping as part of the course?

- ◯ Yes
- ◯ No

Page 04

3. From today's perspective, would you have yourself genotyped?

- ◯ Yes
- ◯ No

4. From today's perspective, would you have yourself genotyped again?

- ◯ Yes
- ◯ No

Page 05

5. Has this course changed your attitude towards personal genotype analysis?

- ◯ Yes
- ◯ No
- ◯ I don't know

Page 06

6. How did the course change your attitude?

- ◯ Through the course I have gained a more positive opinion on genetic analysis
- ◯ Through the course I have become more critical about genetic analysis

Page 07

7. Would you participate in personal genotyping as part of the course if it would be offered via a non-european private company, such as 23andMe?

- ◯ Yes
- ◯ No

Page 08

8. Did you collect the password for your complete genotype data?

- ◯ Yes
- ◯ No

Page 09

9. Do you still plan to collect the password for your complete genomic data?

- ○ Yes
- ○ No

Page 10

10. Did you conduct analyses with your own data beyond the course?

- ☐ Analyzing genetic ancestry
- ☐ Analyzing pharmacogenomic markers (medication effects)
- ☐ Analyzing wellness traits (not disease-relevant, e.g., eye color or blood type)
- ☐ Carrier detection of risk variants for monogenetic diseases
- ☐ Analyzing risk of (common) polygenic diseases
- ☐ No, I just wanted to receive my data

Page 11

11. Do you plan to conduct further analyses with your own genomic data?

- ○ Yes
- ○ No

Page 12

12. Should personal genotyping be allowed without genetic counseling by a medical professional?

- ○ Yes
- ○ No

Page 13

13. Would you recommend personal genotyping to relatives or friends who did not take the course?

- ○ Yes
- ○ No

Page 14

14. How important do you consider the treatment of ethical aspects in the Understanding Your Genome course?

- ○ Absolutely necessary
- ○ Rather important
- ○ Neutral
- ○ Rather unimportant
- ○ Completely unimportant

Page 15

15. Do you think that personal genotyping in the course context is useful for the learning experience?

- ○ Yes
- ○ No

Page 16

16. Would you recommend to offer personal genotyping in future courses again?

- ○ Yes
- ○ No

Page 17

17. Do you feel adequately trained to analyze and interpret genomic data beyond the course?

- ○ Yes
- ○ No

Page 18

18. What would you do differently if you could take the course again and why?

Thank you for your time!

Your answers were transmitted, you may close the browser window or tab now.
