# Supplementary material for: Implementation and evaluation of personal genetic testing as part of genomics analysis courses in German universities
Source: BMC Med Genomics. 2023 Apr 5;16:73. doi: 10.1186/s12920-023-01503-0 (PMC10074719; doi:10.1186/s12920-023-01503-0)
Supplement: Supplementary file 4 — Additional file 4. All conducted questionnaires with survey periods and number of participants. [file 12920_2023_1503_MOESM4_ESM.pdf]

Supplement 4

All conducted questionnaires with the respective survey periods, number of survey participants, number of course participants, and response rates. Additionally, the number of "complete survey participants" who have answered all previous questionnaires in the given academic year is presented for HPI (ST: summer term, WT: winter term).

University	Course year	Course participants	Questionnaire	Survey period	Survey participants (response rate)	Complete survey participants (response rate)
HPI	ST 2020	16	Q1	2020-05-20 – 2020-05-25	15 (94%)	15 (94%)
HPI	ST 2020	16	Q2	2020-07-20 – 2020-07-29	14 (88%)	13 (81%)
HPI	ST 2020	16	Q3	2020-08-11 – 2020-08-27	10 (63%)	8 (50%)
HPI	ST 2020	16	Q4	2020-12-10 – 2020-12-20	10 (63%)	6 (38%)
TUM	All before WT 2020/ 21	<u>Total: 102</u><br>ST 2017: 22<br>WT 2017/18: 26<br>ST 2018: 23<br>WT 2018/19: 13<br>WT 2019/20: 18	Q4'	2020-12-09 – 2020-12-20	<u>Total: 47 (46%)</u><br>ST 2017: 5 (23%)<br>WT 2017/18: 12 (46%)<br>ST 2018: 15 (65%)<br>WT 2018/19: 6 (46%)<br>WT 2019/20: 9 (50%)	–
TUM	WT 2020/ 21	10	Q1	2020-11-18 – 2020-11-23	8 (80%)	–
TUM	WT 2020/ 21	10	Q2	2021-02-08 – 2021-02-15	8 (80%)	–
TUM	WT 2020/ 21	8*	Q3	2021-03-08 – 2021-03-10	7 (88%)	–
TUM	WT 2020/ 21	8*	Q4	2021-05-19 – 2021-05-20	6 (75%)	–
HPI	ST 2021	16	Q1	2021-04-13 – 2021-04-14	16 (100%)	16 (100%)
HPI	ST 2021	16	Q2	2021-07-26 – 2021-08-02	15 (94%)	15 (94%)
HPI	ST 2021	15*	Q3	2021-08-13 – 2021-08-21	9 (60%)	9 (60%)
HPI	ST 2021	15*	Q4	2021-12-06 – 2021-12-16	9 (60%)	6 (40%)

* Not all course participants also participated in the block week
