# Supplementary material for: Implementation and evaluation of personal genetic testing as part of genomics analysis courses in German universities
Source: BMC Med Genomics. 2023 Apr 5;16:73. doi: 10.1186/s12920-023-01503-0 (PMC10074719; doi:10.1186/s12920-023-01503-0)
Supplement: Supplementary file 6 — Additional file 6. Schedule of the Genomic Medicine course at TUM (winter term 2020/21). [file 12920_2023_1503_MOESM6_ESM.pdf]

Supplement 6

Schedule of the Genomic Medicine course at TUM (winter term 2020/21).

Curriculum compulsory optional course Genomic Medicine

School of Medicine, Technical University of Munich, winter term 2020/21

The curriculum in the winter term 2020/21 differed from the usual curriculum because of restrictions on in-person teaching due to COVID-19.

Online lectures ahead of the course

- Session 1: (Common) genetic variation and non-coding genetic variants
- Session 2: Technology of genotyping microarrays, Illumina GSA, genotype calling and accuracy
- Session 3: Informed consent; introduction to different analysis levels: causal disease variants, carrier status, pharmacogenomics, genome-wide association studies, ancestry
- Session 4: NGS sequencing methods, prediction of monogenic disorders
- Session 5: Prediction of genetic risk for breast cancer
- Session 6: Legal and ethical aspects of human genetics with applied examples
- Session 7: Statistics and polygenic scores
- Session 8: Genetic variance in different ancestries, ancestry analyses and admixture
- Session 9: Genome-wide association studies

Main course

Day 1

- Welcome and introduction
- Ancestry analyses (practical introduction, analysis/interpretation of either one's own or openSNP anonymous ancestry analysis results, discussion)
- Introduction to databases for annotating and interpreting genetic variation, introduction to the interpretation of genetic variants, walkthrough examples of genetic analyses
- Initial examination and interpretation of genetic results based on selected variants (either one's own or anonymous openSNP data)
- Q&A on genetic analyses and selection of topics for the presentation on day 3
- Introduction to pharmacogenomics

Day 2

- Genetic counselling (role play/simulation with subsequent discussion)
- Presentation of direct-to-consumer/online/DIY options for genetic analyses
- Continued analysis and interpretation of genetic results on selected variants (either own or anonymous openSNP data) and preparation of presentations for day 3

Day 3

- Student presentations of selected genetic variants and/or genetic disorders (background, disease context, genetic interpretation, effect (size), ancestry dependency, assessment of whether the result is actionable) [evaluated and graded] with subsequent discussion of each presentation
- Introduction to ethics and civil liberties followed by a discussion of ethical aspects of genetic testing
- Discussion on whether direct-to-consumer genetic testing without genetic counselling should be allowed in Germany
- Final Q&A round and feedback
